# Complex bioactive nanofibrous dura mater repairs traumatic brain injury

**DOI:** 10.4103/NRR.NRR-D-25-00621

**Published:** 2025-09-29

**Authors:** Siyu Chen, Xiaopei Zhang, Qingxia Guo, Yuying Yan, Manfei Fu, Yuanfei Wang, Tong Wu

**Affiliations:** 1 The Affiliated Hospital of Qingdao University, School of Basic Medicine, Qingdao Medical College, Qingdao University, Qingdao, Shandong Province, China; 2 Shandong Key Laboratory of Medical and Health Textile Materials, College of Textile & Clothing, Qingdao University, Qingdao, Shandong Province, China; 3 Qingdao Stomatological Hospital Affiliated to Qingdao University, Qingdao, Shandong Province, China

**Keywords:** anti-adhesion, antibacterial, anti-inflammatory, anti-leakage, barrier performance, biocompatibility, differential dual-release, multilayered nanofibrous dura mater, neuroprotection, traumatic brain injury, nerve regeneration

## Abstract

Dura closure following surgery for traumatic brain injury is important to maintain the structural integrity of the brain and serve as a barrier to prevent infection and leakage of cerebrospinal fluid. Although an artificial dural mater can provide barrier capabilities, its performance in the repair of injured neural cells and neuroprotection during the secondary injury stage can be improved. Therefore, we designed and manufactured a multi-layer nanofibrous dura mater containing minocycline and insulin-like growth factor 1 using electrospinning technology to repair tissue following traumatic brain injury. The results showed that the multi-layer nanofibrous dura mater promoted neuronal process transection, hypoxia, and glucose deprivation, as well as survival and neurite extension of SH-SY5Y cells after oxidative stress injury. Minocycline hydrochloride and insulin growth factor 1 were separately incorporated into the fibers to facilitate their differential dual release for immunomodulation during the early stage of traumatic brain injury and provide neuroprotection during the repair process. In addition, the multi-layered nanofibrous dura mater promoted the increase in M2 polarization for microglia and the secretion of anti-inflammatory cytokines, which enhanced neural cell survival. Furthermore, we verified the antibacterial, anti-adhesion, barrier performance, anti-leakage, and biocompatibility capabilities of the multi-layered nanofibrous dura mater. Therefore, our multi-layered nanofibrous dura mater containing minocycline and insulin-like growth factor 1 has great potential as a substitute for dura mater and promotes nerve recovery following traumatic brain injury.

## Graphical abstract

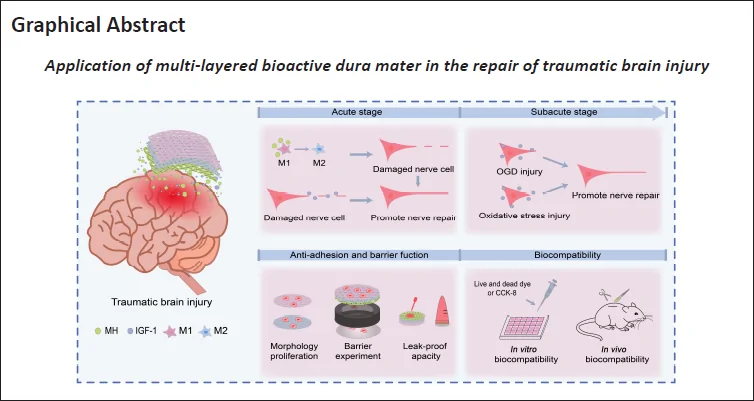

## Introduction

Traumatic brain injury (TBI) is a type of brain injury caused by various factors, such as the head being hit by a blunt object, violent impact, or penetrating injury to the head (Savelieff and Feldman, 2021; Lang et al., 2023). Such damage can lead to extensive physical and functional disorders, including significant physical decline, cognitive decline, and mood swings (Howlett et al., 2022; Delmonico et al., 2024). TBI disrupts the normal functions of neural cells and threatens their survival. Moreover, it can disrupt synaptic circuits and highly organized brain network systems, which impacts an individual’s daily life and social functioning (Zhao et al., 2023; Xu et al., 2024; Zhou et al., 2024). The dura mater is located between the skull and brain tissue and is a natural barrier that covers the brain surface. Following TBI, the dura mater can be damaged, which can hinder this natural barrier function (Khurana et al., 2024). Owing to the limited self-repair mechanism of the dura mater, timely surgical repair is necessary to restore its integrity, prevent abnormal loss of cerebrospinal fluid (CSF), minimize the risk of infection, and ensure the support and conditions needed for the natural healing of the affected brain area (Qi et al., 2025). Defects of the dura disrupt the homeostasis of CSF, which results in leakage of CSF and an increase in the risk of secondary brain injury (Cheng et al., 2024). The activation of fibroblasts after dural injury promotes the secretion of the extracellular matrix (ECM) to promote wound repair. However, overproduction of ECM can lead to pathological fibrosis and tissue adhesion, which can disrupt the recovery of damaged nerves and functions (Dorrier et al., 2021; Feng et al., 2023; Younesi et al., 2024). Common substitutes for the dura mater in clinical practice are limited by their insufficient mechanical properties, inability to effectively inhibit acute inflammatory responses, and lack of long-term release of regenerative factors. This has led to the limited efficacy of currently available clinical dura mater in addressing the constantly changing repair needs following TBI. Therefore, there is an urgent need to develop a multifunctional dura mater that meets the complex demands of TBI repair.

The rapid development of biomedical materials has led to a considerable rise in applications of biomedical scaffolds for the treatment of various diseases (DeBrot and Yao, 2018; Zhang et al., 2019). Recently, electrospinning technology has been widely applied to tissue repair and regenerative medicine (Chen et al., 2025; Shi et al., 2025). This technology allows precise control of the diameter and morphology of the fibers by tuning the electrospinning parameters. This flexible and controlled structural design enables the incorporation of various fiber morphologies, such as porous and core-shell structures (Shahriar et al., 2019; Zhou et al., 2025), and significantly enhances the biological function of the scaffold by mimicking the microenvironment of the ECM, thereby improving the overall performance of the scaffold (Liao et al., 2021; Wang et al., 2022). However, the majority of electrospun artificial dura mater developed to date exhibit functional limitations. For example, although some electrospun artificial dura mater are effective in preventing tissue adhesions, their anti-leakage properties remain inadequate (Zhao et al., 2022). In contrast, other electrospun dura mater have good anti-leakage properties, yet their biological activity is insufficient for promoting neural tissue repair (Sun et al., 2024). Crucially, a traditional electrospun dura mater, based on a single drug delivery system, does not satisfy the nerve repair needs at different stages following TBI. Therefore, currently available electrospun dura mater has limited efficacy in the treatment of neurological dysfunction after TBI.

Therefore, we aimed to develop a multifunctional dura that meets the complex needs of repair following TBI. Lu et al. (2022) and Mohebali and Abdouss (2020) demonstrated the prominent anti-inflammatory effects and antibacterial properties, respectively, of minocycline hydrochloride (MH). Insulin-like growth factor 1 (IGF-1) induces a neuroprotective effect by effectively inhibiting neuronal apoptosis and promoting neural cell survival and regeneration (Bhalla et al., 2022; Hanwright et al., 2022). Given the above findings, we aimed to develop a dual-layered core-shell structured dura mater with MH and IGF-1 using electrospinning techniques. The distinct release profiles of two drugs over time will promote nerve repair after brain injury.

To further enhance the multiple functions of the dura mater, such as anti-leakage and anti-adhesion, we designed two additional layers of dura mater, in which this inner layer formed a multi-layered nanofibrous dura mater (MNDM). The middle nanofibrous dura mater (NDM) layer was composed of polycaprolactone (PCL) nanofibers, which effectively prevents CSF leakage following TBI. Additionally, we introduced hyaluronic acid (HA) to prevent excessive fibroblast proliferation and reduce the risk of adhesion (Ding et al., 2024; Feng et al., 2024).

To verify whether the MNDM meets the repair needs of complex pathological environments after TBI and promotes the repair and regeneration of damaged nerves, we evaluated the MNDM using a variety of experimental techniques: nerve injury, anti-inflammatory, and anti-adhesion assays.

## Methods

### Fabrication and characterization of the nanofibrous dura mater

#### Fabrication of the nanofibrous dura mater

For the preparation of the outer NDM, PCL (Mw 8 × 10^4^, Sigma-Aldrich, St. Louis, MO, USA, Cat# 440744) was added to hexafluoroisopropanol (HFIP; Shanghai Dare Science and Technology Co., Ltd, Shanghai, China, Cat# 920-66-1-CBB) at a mass volume ratio of 10% (w/v), and HA (Mw 0.8–1.5 × 10^6^, Macklin, Shanghai, China, Cat# H909938) was added to formic acid (Sinopharm Chemical Reagent Co, Shanghai, China, Cat# 10010118) at a mass volume ratio of 2% (w/v). After the two solutions were thoroughly stirred until complete dissolution occurred, the PCL and HA solutions were mixed at a volume ratio of 10:1. Subsequently, 1% Span-80 was added to form a PCL/HA spinning solution. Nanofiber membranes were prepared using an electrospinning device (Beijing Yongkang Leye Science and Technology Development Co., Ltd., Beijing, China), which generated PCL/HA random nanofiber membranes. The outer NDM (PCL/HA-NDM) was then obtained. The electrospinning parameters were as follows: positive voltage of 10–15 kV, flow rate of 3 mL/hour, duration of 40 minutes, and receiving distance of 20 cm.

For the preparation of the middle NDM, PCL was added to HFIP at a mass volume ratio of 10% (w/v) and stirred thoroughly using a magnetic stirrer until complete dissolution of the PCL was achieved to obtain a PCL/HA spinning solution. The PCL random nanofiber membrane was prepared using an electrospinning device to produce a middle NDM (PCL-NDM). The electrospinning parameters were as follows: positive voltage of 10–15 kV, flow rate of 3 mL/hour, duration of 40 minutes, and receiving distance of 20 cm.

For the preparation of the inner NDM, gelatin (GEL; Sigma-Aldrich, Cat# V900863) and PCL were added to HFIP at a mass ratio of 7:3 at a concentration of 8% (w/v). MH (Macklin, Cat# M812977) was incorporated at a concentration of 1 mg/mL and stirred thoroughly with a magnetic stirrer until it was completely dissolved to obtain a mixed solution of PCL/GEL/MH. This served as the shell solution. The core layer solution was composed of an aqueous phase containing IGF-1 (Bioss, Beijing, China, human, Cat# BS-0014P), bovine serum albumin (BSA; Solarbio, Beijing, China, Cat# A8020), and the oil phase containing the PCL solution. We prepared aqueous solutions of IGF-1 and BSA at a concentration of 1 mg/mL. PCL was added to dichloromethane (Macklin, Cat# D116144) at a mass volume ratio of 8% (w/v). We mixed the PCL solution with the IGF-1/BSA aqueous solution at a volume ratio of 20:1, then added Span-80 solution (1% [v/v], Solarbio, Cat# S5260). The mixture was stirred with a magnetic stirrer for 6 hours to obtain an emulsion electrospinning solution of IGF-1/BSA/PCL for the core layer solution. The IGF-1/BSA/PCL/GEL/PCL/MH dura mater was prepared using coaxial electrospinning equipment (Beijing Yongkang Leye Technology Development Co., Ltd., Beijing, China). This process resulted in the formation of an inner NDM containing MH and IGF-1 (MH + IGF-NDM). The electrospinning parameters were as follows: positive voltage of 10–15 kV, flow rates of 0.5 mL/hour for the core layer and 1.5 mL/hour for the shell layer, duration of 1 hour and 10 minutes, and receiving distance of 15 cm.

Preparing the MNDM repair scaffolds involved depositing the three-layered scaffolds layer by layer. The resulting three-layered scaffolds were then crosslinked via glutaraldehyde fumigation in a pot for 4 hours to obtain the MNDM containing MH and IGF-1 (MH + IGF-MDNM).

#### Morphology of the nanofibrous dura mater

Cross-sections of each layer of the NDM (i.e., outer, middle, and inner) and the MDNM were photographed for observation using a scanning electron microscope (Regulus 8100, Hitachi Scientific Instruments Co., Ltd., Beijing, China) and a camera (Nikon D5600, Nikon Co., Ltd., Japan). We randomly selected 120 fibers, and their average diameter was measured using ImageJ (version 1.8.0, National Institutes of Health, Bethesda, MD, USA) (Schneider et al., 2012).

#### Contact angle and dual core-shell structure

The hydrophilicity and hydrophobicity of the NDM were measured at room temperature using a contact angle analyzer (OCA 25, DataPhysics Company, Stuttgart, Germany). To validate the MH + IGF-NDM nanofibers with a dual core-shell structure, the aqueous phase of the emulsion was first labeled with fluorescein isothiocyanate (FITC; green fluorescence), and the core-shell structure of the nanofibers prepared using the emulsion electrospinning technique was observed by fluorescence microscopy (ECHO microscopes, ECHO Laboratories, San Diego, CA, USA). The core-shell structure of the prepared fibers was then observed by fluorescence microscopy using FITC-labeled core emulsion (green fluorescence) and rhodamine-labeled shell solutions (red fluorescence). To further verify the core-shell structure of the prepared fibers, the structure was further observed using a transmission electron microscope (JEM-1400Flash, Nippon Electronics Co., Tokyo, Japan).

#### In vitro drug release studies

The MH + IGF-NDM was prepared as described previously and cross-linked with glutaraldehyde for 4 hours. We then placed 50 mg of dura mater in a centrifuge tube containing 5 mL of phosphate-buffered saline (PBS), which was shaken at 120 revolutions/minute (rpm) in a temperature-controlled shaker at 37°C to simulate *in vivo* physiological conditions. At predetermined time points (1, 2, 3, 4, and 12 hours and 1, 2, and 3 days), 1 mL of the solution sample was removed to measure its absorbance wavelength at 360 nm using a microplate reader (Thermo Scientific Varioskan LUX) to monitor the release of MH. We used rhodamine B (RB) as a drug model (Zhang et al., 2011; Park et al., 2023) to study the drug release behavior in the core layer of the dural stent. The preparation parameters of the MH + RB-NDM were the same as those described above, except that RB at a dose of 1 mg/mL was encapsulated instead of IGF-1. For each group, 50 mg of dura mater was weighed and added to centrifuge tubes containing 5 mL of PBS. The tubes were placed in a constant temperature shaker at 37°C and shaken at 120 rpm. A 1-mL sample of the solution was extracted at predetermined time points (1, 3, 5, 7, 10, 14, 20, 27, 35, and 42 days). The absorbance at 554 nm was measured using a microplate reader to monitor the release curve of RB, which indirectly reflects the release characteristics of IGF-1. After each sample was obtained, an equal volume of fresh PBS was added to the centrifuge tube.

Before conducting the drug release study, the loading efficiency of MH was determined by calculating the ratio of MH encapsulated in the MH + IGF-NDM to the initially added MH quantity. The dura mater was dissolved in 11 mL of HFIP and shaken for 2 minutes, and the optical density of the solution at 360 nm was measured. For RB, the loading efficiency was determined by the ratio of RB in the MH + RB-NDM to the initial amount of RB. The dura mater was dissolved in 2 mL of dichloromethane with 1 minute of shaking, and the optical density of the solution at 554 nm was measured. The loading efficiency was calculated as follows:


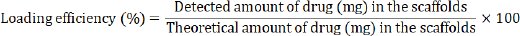


#### Mechanical properties

The mechanical properties of the dura mater were tested using a microcomputer-controlled electronic universal testing machine (WDW-5G, Jinan Hengshishengda Co., Ltd., Jinan, Shandong, China). The PCL/HA-NDM, PCL-NDM, MH + IGF-NDM, and MH + IGF-MDNM were cut into 0.5 cm × 5 cm rectangles, and the thickness of each specimen was measured using a screw micrometer (Deli Company, Ningbo, Zhejiang, China). Tests were conducted at a tensile speed of 5 mm/minute until breakage occurred, with three specimens tested for each membrane layer.

#### In vitro degradation study

The weight change, pH, and microstructure of the MH + IGF-MDNM were analyzed at different time points after immersion in PBS (pH = 7.35). The prepared scaffolds (50 mg, three samples per group) immersed in PBS were placed in a shaker at 37°C and 120 rpm. The MH + IGF-MDNM was removed and dried at predetermined time points (10, 20, 30, 60, 90, 120, 150, and 180 days). The pH of PBS and the weight of the MH + IGF-MDNM were measured, and the dried MH + IGF-MDNM samples collected on days 30, 60, and 90 were analyzed for microstructure using a scanning electron microscope.

### Evaluation of the nerve injury model

Human neuroblastoma cells (SH-SY5Y cells; Institute of Nerve Rehabilitation and Regeneration, Qingdao University, Cat# CL-0208, RRID: CVCL_0019) were cultured in Dulbecco’s modified Eagle medium (DMEM; Solarbio, Cat# 11995), supplemented with 10% fetal bovine serum (PAN-Biotech, Edenburg, Bavaria, Germany, Cat# ST30-3302) and 1% penicillin-streptomycin (Biosharp, Hefei, Anhui, China, Cat# BL505A), and placed in an incubator at 37°C with 5% carbon dioxide (CO_2_).

#### Nerve neurite transection model

The neurite transection model was used to simulate the effects of primary neural injury after TBI. SH-SY5Y cells (6 × 10^3^ cells/well) were cultured for 24 hours. Then, 20 transverse and vertical incisions were made in each well to scratch the SH-SY5Y cells and transect their neurites, simulating a nerve injury. Cells that underwent no neurite transection treatment served as controls. Cell viability was assessed before and after neurite transection using the cell counting kit-8 (CCK-8; Glpbio, Monteclair, CA, USA, Cat# GK10001). The CCK-8 solution (10%, comprising 40 μL of CCK-8 mixed with 360 μL of DMEM) was added to each well and incubated for 90 minutes at 37°C. At the end of the incubation, the absorbance value of each well was determined at 450 nm. Additionally, SH-SY5Y cells were stained 1 day after the neurite transection treatment using a neurite outgrowth staining kit (Thermo Fisher, Waltham, MA, USA, Cat# A15001). The cells were observed using a fluorescence microscope, and the neurite length of cells was measured in 120 randomly selected cells from each group using ImageJ (Morrison et al., 2011; Wang et al., 2022).

#### Oxygen and glucose deprivation injury model

The oxygen and glucose deprivation (OGD) injury model was used to simulate secondary neural injury after TBI. SH-SY5Y cells (6 × 10^3^ cells/well) were cultured for 24 hours. Subsequently, normal medium was replaced with deoxygenated glucose-free extracellular solution, and the well plates were placed in sealed anaerobic chambers containing anaerobic bags (Thermo Fisher, Cat# AN0025A) and incubated at 37°C with 5% CO_2_ atmosphere for 3 hours, after which the medium was reverted to normal culture conditions. Cell viability was assessed before and after OGD treatment using the CCK-8. SH-SY5Y cells before and after OGD treatment were stained with a neurite outgrowth staining kit, and the neurite length of the cells was measured in 120 randomly selected cells from each group using ImageJ (Zhao et al., 2022; Yuan et al., 2023).

#### Oxidative stress injury model

The oxidative stress injury model was used to simulate secondary neural injury after TBI. SH-SY5Y cells (6 × 10^3^ cells/well) were cultured for 24 hours. Oxidative stress injury was induced in SH-SY5Y cells using different concentrations of hydrogen peroxide (H_2_O_2_) for 24 hours. CCK-8 was used to identify an appropriate H_2_O_2_ stimulation concentration, which was subsequently used to treat SH-SY5Y cells to produce oxidative stress. The H_2_O_2_-treated SH-SY5Y cells were stained with a neurite outgrowth staining kit, and the neurite length of the cells was measured in 120 randomly selected cells from each group using ImageJ (Zhang et al., 2021; Tie et al., 2022).

### Evaluation of neural cell viability and neurite regeneration

#### Acute-phase nerve injury repair study

SH-SY5Y cells (6 × 10^3^ cells/well) were cultured for 24 hours. An acute nerve injury model was established via neurite transection or oxidative stress induction. Five groups were established for the experiment: control group, dura mater group without drug (NDM group), dura mater group loaded with MH (MH-NDM group), dura mater group loaded with IGF-1 (IGF-NDM group), and dura mater group loaded with both MH and IGF-1 (MH + IGF-NDM group). We formed a co-culture system with cells treated in the lower chamber by placing the dura mater (the diameter of the circular NDM was 14 mm) of each group into the upper chamber of the Transwell (Labselect, Tianjin, China). Cells were co-cultured for 1, 3, and 5 days. The dura mater of each group was double sterilized using 75% ethanol fumigation for 4 hours and ultraviolet irradiation for 30 minutes. Cell viability was measured using CCK-8 on days 1, 3, and 5 after neurite transection or oxidative stress induction. To further observe the effect of nerve repair, we used a neurite outgrowth staining kit to perform immunofluorescence staining of cells 3 days after neurite transection or oxidative stress induction. The cells were observed under a fluorescence microscope, and the neurite length of the cells was measured in 120 randomly selected cells from each group using ImageJ.

#### Subacute-phase nerve injury repair study

SH-SY5Y cells were seeded at a density of 6 × 10^3^ cells/well in 24-well plates and cultured for 24 hours. SH-SY5Y cells were subjected to OGD or oxidative stress induction to mimic the pathological process of subacute nerve injury. The experimental design comprised the same five groups as those used in the acute phase experiment. The extracts of different dura mater that had been incubated with normal medium for 14 days were collected ahead of time and diluted with fresh medium at a ratio of 1:1 to generate conditioned medium for subsequent co-culture experiments. Assessment of changes in cell viability, immunofluorescence staining, and measurement of neurite length were the same as those used in the acute-phase studies.

To further investigate the effect of different dura mater nanofibers on the apoptosis of damaged nerves, SH-SY5Y cells were inoculated into 12-well plates (1 × 10^5^ cells/well), treated with OGD, and co-cultured with different dura mater for 3 days. Cells were collected via centrifugation at 300 × *g* for 5 minutes, washed twice with PBS solution, and treated using the Annexin V-FITC/PI apoptosis kit according to manufacturer instructions (Elabscience, Wuhan, Hubei, China, Cat# E-CK-A211). The effect of different dura mater on the apoptosis of SH-SY5Y cells subjected to OGD was detected using flow cytometry (Thermo Fisher) and analyzed using Flowjo software (version 10.9.0, Ashland, OR, USA).

### Anti-inflammatory evaluation

Murine microglial cells (BV2 cells, Institute of Nerve Rehabilitation and Regeneration, Qingdao University, Cat# STCC20009G, RRID: CVCL_0182) were used to establish an inflammation model by treating BV2 cells with 1 μg/mL lipopolysaccharide (LPS; Sigma-Aldrich, Cat# L3012) for 24 hours (Lee et al., 2018). BV2 cells were cultured in DMEM, supplemented with 10% fetal bovine serum and 1% penicillin-streptomycin, and placed in an incubator at 37°C with 5% CO_2_. BV2 cells (5 × 10^3^ cells/well) were cultured for 24 hours. The cells were assigned to five groups: control group without LPS induction, LPS group, LPS + dura mater without drug group (NDM group), LPS + MH-loaded dura mater (MH-NDM group), and LPS + MH drug (MH group). Different types of dura mater were placed in the upper chamber of the transwell, and a co-culture system with LPS-treated BV2 cells was established in the lower chamber. Cell viability was assessed using CCK-8 1 day after the different dural interventions. Additionally, the release of nitric oxide (NO) was quantified in each experimental group using a NO assay kit (Beyotime, Shanghai, China, Cat# S0021S). For immunofluorescence staining, cells were fixed in 4% paraformaldehyde, treated with 0.25% Triton X-100, and blocked in 1% BSA. Subsequently, cells were incubated separately with inducible nitric oxide synthase (iNOS; rabbit, 1:250, Abcam, Cambridge, UK, Cat# ab3523, RRID: AB_303872) and arginase-1 (goat, 1:250, Abcam, Cat# ab60176, RRID: AB_943473) at 4°C overnight. After washing, cells were incubated separately with goat anti-rabbit immunoglobulin G (IgG) H&L (Alexa Fluor 594, 1:500, Abcam, Cat# ab150080, RRID: AB_2650602) and donkey anti-goat IgG H&L (Alexa Fluor 488, 1:500, Abcam, Cat# ab150129, RRID: AB_2687506) in the dark at 37°C for 1 hour. The cells were washed again and stained with 4′,6-diamidino-2-phenylindole (DAPI; Solarbio, Cat# S2110) for 10 minutes. Finally, the immunofluorescence expression of the cells in the different groups was observed using a fluorescence microscope. Twelve images from each group were randomly selected to quantify fluorescence intensity using ImageJ.

### Evaluation of modulating inflammatory cell polarization for nerve regeneration

To investigate the effect of the dura mater on nerve regeneration via the modulation of BV2 polarization, we set up seven groups: control group using a normal medium, BV2 group, LPS group, LPS + dura mater without drug group (NDM group), LPS + dura mater loaded with MH group (MH-NDM group), LPS + dura mater loaded with IGF-1 group (IGF-NDM group), and LPS + dura mater loaded with MH and IGF-1 group (MH + IGF-NDM group).

BV2 cells were seeded at a density of 5 × 10^3^ cells/well in 24-well plates for all experimental groups (BV2, LPS, NDM, MH-NDM, IGF-1-NDM, and MH + IGF-1-NDM) and cultured for 24 hours. All groups, apart from the BV2 group, were subjected to LPS (1 μg/mL) stimulation to induce an inflammatory response. Subsequently, different types of dura mater were placed in the upper chamber of the transwell to create a co-culture system, with the LPS-treated BV2 cells in the lower chamber. On day 2 of the dura mater intervention, DMEM was replaced to continue the culture for an additional day, and the medium from each group was collected and mixed at a 1:1 ratio with DMEM to prepare the conditioned medium.

SH-SY5Y cells (6 × 10^3^ cells/well) were cultured for 24 hours. Subsequently, cells were subjected to neurite transection or OGD to establish a nerve injury model. The conditioned medium was used for co-culturing with the injured SH-SY5Y cells. Cell viability was assessed after 3 days of co-culture using the CCK-8.

### Antibacterial evaluation

The antibacterial rate of the dura mater against *Staphylococcus aureus* (*S. aureus*, ATCC, Hangzhou, China, 43300) and *Escherichia coli* (*E. coli*, ATCC, 700928) was calculated by comparing the rate with that of the control group. The nutrient broth was prepared by mixing 0.6 g of beef powder (Aobox, Beijing, China, Cat# 01-008), 1 g of peptone (Aobox, Cat# 01-001), and 200 µL of deionized water. The solid agar culture medium was prepared by mixing 0.9 g of beef powder, 1.5 g of peptone, 4.5 g of agar powder (Aobox, Cat# 01-024), and 300 µL of deionized water.

The latter medium was introduced to the Petri dish after pressure steam sterilization. Bacteria were inoculated in the broth and incubated at 200 rpm for 24 hours at 37°C. The concentration of the bacteria was adjusted to 1 × 10⁵ colony-forming units (CFU)/mL according to the gradient dilution method with PBS. Subsequently, 0.15 g of the NDM was fumigated with alcohol, sterilized with ultraviolet light, and co-cultured with bacterial solution in a constant-temperature shaker at 24°C and 150 rpm for 24 hours. Then, 100 µL of bacterial solution was evenly coated on agar plates, and the number of colonies was counted after incubation at 37°C for 24 hours. The number of colonies in the blank control group was defined as A0, and the number of colonies in the experimental group was defined as A1. The antibacterial rate was calculated using the formula:


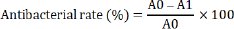


### Anti-adhesion evaluation

Murine embryonic fibroblast cells (NIH3T3 cells, Institute of Nerve Rehabilitation and Regeneration, Qingdao University, Cat# TCM-C752, RRID: CVCL_0594) were cultured in DMEM, supplemented with 10% fetal bovine serum and 1% penicillin-streptomycin, and subsequently placed in an incubator at 37°C with 5% CO_2_. A bioadhesive was used to secure the prepared PCL-NDM and PCL/HA-NDM to 24-well-sized slides, and an empty blank slide group served as the control. All slides and materials were sterilized using 75% ethanol fumigation for 4 hours and ultraviolet irradiation for 30 minutes before cell inoculation. NIH3T3 cells were seeded at a density of 6 × 10^3^ cells/well in 24-well plates for all groups. The CCK-8 was used to assess the number of NIH3T3 cells adhering to the different types of NDM at 6 and 12 hours after inoculation and cell proliferation at 1, 3, and 5 days post-inoculation.

We evaluated the effects of the different dura mater on cell proliferation using Ki67 (rabbit, Affinity Biosciences, Changzhou, Jiangsu, China, Cat# AF0198, RRID: AB_2834152). Cells were fixed with 4% paraformaldehyde, permeabilized with 0.25% TritonX-100, and blocked with 5% BSA. Ki67 (1:200) was then incubated at 4°C overnight. After washing, cells were treated with Alexa Fluor 594 (1:500) at 37°C for 1 hour, rewashed, and stained with DAPI. Next, the immunoreactivity of the different cell groups was observed using a fluorescence microscope. A total of nine images were randomly selected from each group of samples, and quantitative analysis of fluorescence intensity was performed using ImageJ.

To examine the cell morphology of each group, cells were fixed with 4% paraformaldehyde, treated with 0.1% TritonX-100, and blocked with 1% BSA at room temperature. After adding Phalloidin-iFluor 488 dye (1:2000, Abcam, Cat# ab176753) at 37°C for 30 minutes, cells were washed with PBS and stained with DAPI. The morphology of cells was examined using a fluorescence microscope.

### Evaluation of barrier performance

Five groups were established: medical gauze group (control group), PCL/HA-NDM group, PCL-NDM group, MH + IGF-NDM group, and MH + IGF-MNDM group. Membranes were cut into 14-mm-diameter circles and attached to a steel ring (with a diameter of 14 mm), which was then placed in a 24-well plate, ensuring that the membrane surface of each group did not touch the bottom of the 24-well plate. NIH3T3 cells (6 × 10^3^ cells/well) were cultured for 3 and 5 days, after which the steel ring was removed. The top surface of the dura mater, the bottom surface of the dura mater, and the well plate bottom were each fixed with 4% paraformaldehyde and stained with DAPI. Cells were observed using a fluorescence microscope and photographed to examine whether they had penetrated the membrane and reached the bottom of the wells. The number of cells at the bottom of the well plates was then counted in nine randomly selected fluorescence images from each group and analyzed using ImageJ.

### Anti-permeability evaluation

Four groups were established: PCL/HA-NDM group, PCL-NDM group, MH + IGF-NDM group, and MH + IGF-MNDM group. For the pressure-free droplet infiltration experiment, the dura mater of each group was cut into 14-mm-diameter discs and fixed onto a clean surface. Next, 5 μL of RB solution (concentration 0.25 mg/mL) was applied to each distinct dura mater. After depositing the RB solution onto the dura mater, the penetration of the RB droplets from one side of the material to the other was examined in each group.

Fluorescent dye penetration experiment: MH + IGF-MDNM was cut into 14-mm-diameter circles and affixed to a clean surface. Subsequently, 10 μL of RB (concentration 0.25 mg/mL) was placed on the membranes. After adding the RB solution, RB droplets were examined after 5, 10, 30, and 60 minutes using fluorescence microscopy to determine whether they had penetrated from one side to the other.

For the inverted centrifugal tube penetration experiment, we added 1 mL of RB (concentration 0.25 mg/mL) to different 1.5-mL centrifuge tubes to assess the ability of different meninges to prevent leakage of static solutions. The cut PCL/HA-NDM, PCL-NDM, MH + IGF-NDM, and MH + IGF-MNDM were attached to the mouths of these tubes with bioadhesive and inverted. We observed the leakage of droplets into the mouth of the tube at different times and acquired photographs. To further observe the anti-leakage ability of different membranes in dynamic aqueous solutions, 15 mL of aqueous solution was added to a 15-mL centrifuge tube, and the mouth of the tube was sealed with the four different cerebral membranes mentioned above. The tubes from each group were then inverted and vigorously shaken for a minimum of 30 seconds, and the membranes’ anti-leakage ability in dynamic conditions was assessed.

### Biocompatibility experiments

#### In vitro biocompatibility experiments

SH-SY5Y and NIH3T3 cells (5 × 10^4^ cells/well) were cultured for 24 hours. Five groups were established: control group, MNDM group, MH + MNDM group, IGF-MNDM group, and MH + IGF-MNDM group. A co-culture system was created using a transwell chamber. Before cell inoculation, all dura mater underwent double sterilization. On day 2, a live/dead staining kit (Solarbio, Cat# CA1630) was used to perform the staining procedure. Calcein acetoxymethyl ester (1:2000) and propidium iodide (1.5:2000) were incubated for 20 minutes under conditions that prevented light exposure. The staining results were examined using a fluorescence microscope. The number of viable and dead cells in 15 randomly selected images from each group was analyzed using ImageJ. Both SH-SY5Y and NIH3T3 cells (5 × 10^3^ cells per well) were cultured for 24 hours. After 3 days of co-culture, the assay was conducted using the CCK-8.

#### In vivo biocompatibility experiments

For the subcutaneous implantation experiment, six male Sprague–Dawley rats (aged 6–8 weeks, body weight ranging from 200–250 g) were purchased from Peng Yue Co., Ltd. (Jinan, Shandong, China; license No. SCXK [Lu] 20220006). All animal care and experiments were approved by the Animal Ethics Review Committee of The Affiliated Hospital of Qingdao University (approval No. AHQU-MAL20241101CSY, November 1, 2024). All experiments were designed and reported according to the Animal Research: Reporting of *In Vivo* Experiments guidelines (Percie du Sert et al., 2020).

The animals were raised in the SPF-level Animal Experimental Center of The Affiliated Hospital of Qingdao University in an environment with a temperature of 23 ± 2°C and a 12-hour light-dark cycle. The experiment comprised five groups: control group (sham operation group), PCL/HA-NDM group, PCL-NDM group, MH + IGF-NDM group, and MH + IGF-MNDM group. Experiments were conducted at two time points: 3 and 7 days after injury. Three rats were arbitrarily assigned to each time point (*n* = 3/time point). The different dura mater of each group was implanted into the back of each rat, ensuring three parallel samples per group per time point (*n* = 3 in each group/time point).

The Sprague–Dawley adult male rats were examined and acclimated to the environment for 5 days. Rats were anesthetized with isoflurane (Rivord Life Technology Co., Ltd., Shenzhen, Guangdong, China) using an animal anesthesia machine (R500IE, RWD, Rweid Life Technology Co., Ltd., Shenzhen, Guangdong, China). The rats were placed in the induction box of the anesthesia machine and anesthetized with 4%–5% isoflurane for 4–5 minutes. The rats were then transferred to the operating table and connected to a nasal mask, inhaling 2%–3% isoflurane (oxygen flow rate 0.5–1 L/min). During the experiment, the concentration of isoflurane was adjusted according to the anesthesia status of the rat. Before implantation, the dura mater (0.5 cm × 0.5 cm in size) in each group was sterilized and immersed in sterile saline solution. Five incisions approximately 0.8 cm long were made on the back of each rat, and the subcutaneous tissue was gently separated to facilitate the implantation of the different samples. On days 3 and 7 after surgery, the animals were sacrificed by cervical dislocation to obtain the implants and surrounding tissues. The samples were fixed in 4% paraformaldehyde for 24 hours, dehydrated, embedded in paraffin, and sectioned for hematoxylin & eosin and Masson’s staining (Zhuhai Baso Biotechnology Co., Ltd., Zhuhai, Guangdong, China).

### Traumatic brain injury experiment

For the TBI experiment, 18 male Sprague–Dawley rats aged 6–8 weeks and weighing between 200–250 g were purchased from Peng Yue Co., Ltd. All animal care and experiments were approved by the Animal Ethics Review Committee of The Affiliated Hospital of Qingdao University (approval No. AHQU-MAL20250321CSY, March 21, 2025). All the TBI rats were raised in the SPF-level Animal Experimentation Center of The Affiliated Hospital of Qingdao University. They were randomly divided into the sham surgery group (sham group), TBI group, or TBI + MH + IGF-MNDM group (*n* = 6 in each group).

The rats were observed and allowed to acclimate to their environment for 7 days. Sprague–Dawley rats were then anesthetized with isoflurane using the same procedure described above. Rats were placed in a stereotaxic apparatus (Beijing Zhongshi Dichuang Technology Development Co., Ltd., Beijing, China) in a prone position, and the scalp was incised along the midline to expose the skull. A 5-mm-diameter round bone window was cut in the right parietal region (3 mm posterior to the bregma and 3 mm adjacent to the midline) using a sterile drill bit to expose the brain tissue. Trauma was applied using a TBI impact device: a 40-g impact weight was dropped freely from a height of 20 cm, with a needle tip diameter of 3 mm, and the depth of impact was accurately controlled to 3 mm. The sham group underwent craniotomy without impingement. All MH + IGF-MNDM (0.5 cm × 0.5 cm in size) were sterilized before implantation, followed by precise placement of the dura mater at the trauma site, bone wax closure, and final suturing of the scalp.

After the operation, animals were placed in incubators for recovery, and vital signs were monitored continuously. On postoperative day 3, the brain tissues of the rats in each group were obtained immediately after anesthesia. The brain tissues were placed in pre-cooled PBS, completely homogenized using a tissue homogenizer, and centrifuged at 5000 × *g* for 5 minutes at 4°C. The supernatants were collected for immediate assay. The expression levels of inflammatory factors were quantified using enzyme-linked immunosorbent assays with rat tumor necrosis factor-α (TNF-α; Multiscience, Hangzhou, Zhejiang, China, Cat# EK382/3-96) and rat interleukin-1β enzyme-linked immunosorbent assay kits (Multiscience, Cat# EK301B/3-96), respectively.

### Statistical analysis

Statistical analysis was performed using the GraphPad Prism 10.1.2 software (GraphPad Inc., San Diego, CA, USA, www.graphpad.com). We used independent samples *t*-tests or one-way analysis of variance followed by Tukey’s multiple comparison test. A sample size of *n* ≥ 3 animals per group was maintained to meet the conventional standards of animal experimental research. Animals that died were not included in the statistical analyses. The evaluators were informed of the animal groupings. All experiments were performed using at least three parallel samples. Statistical significance was denoted as *P* < 0.05. Data are presented as means ± standard deviations.

## Results

### Characterization of the nanofibrous dura mater

**Figure 1:**
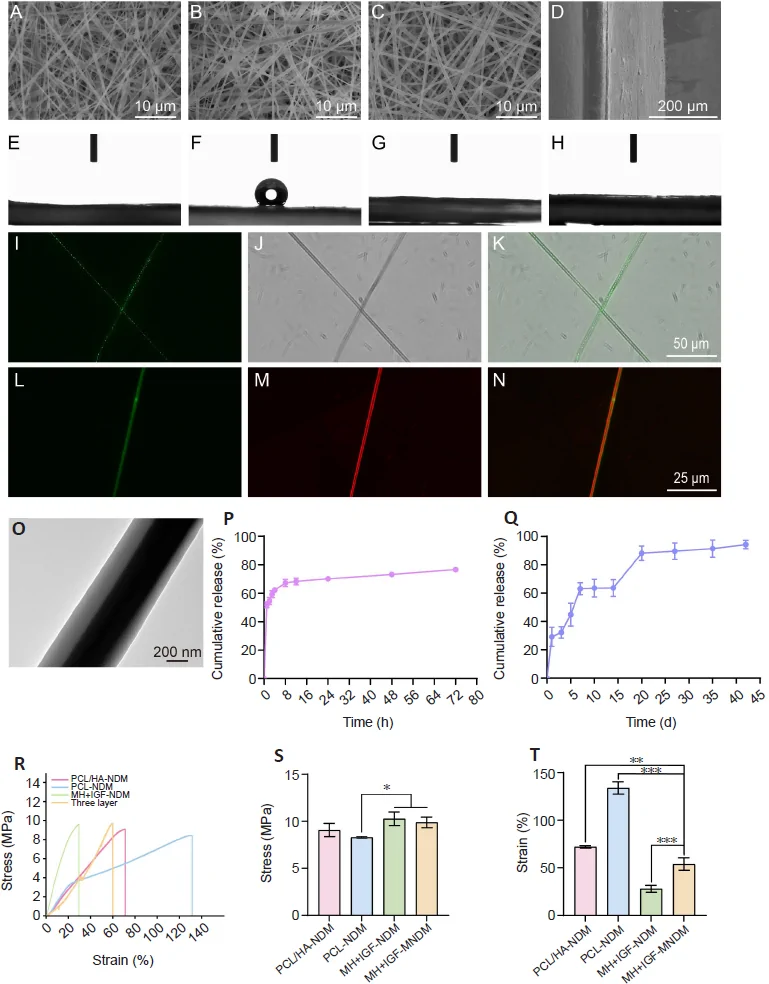
Characterization of NDM: structure, hydrophilicity and hydrophobicity, drug release, and mechanical properties. (A–D) Images of each layer of the PCL/HA-NDM (A), PCL-NDM (B), and MH + IGF-NDM (C), and cross sections of the MH + IGF-MNDM (D). The fibers in each layer of dura mater were randomly arranged, forming an interwoven fiber network structure. (E–H) The water contact angle of each layer of the PCL/HA-NDM (E), PCL-NDM (F), MH + IGF-NDM (G), and MH + IGF-MNDM (H). (I–N) The core-shell structure of the emulsion nanofibers (I–K) and emulsion combined with coaxial nanofibers (L–N) (*n* = 3). (O) Transmission electron microscopy image of the MH + IGF-NDM. Scale bars: 10 μm (A–C), 200 μm (D and O), 50 μm (I–K), and 25 μm (L–N). (P and Q) Sustained release curves for MH (P) and RB (Q) for the MH + IGF-NDM (*n* = 4). (R–T) Tensile stress-strain curve (R), ultimate strength (S), and elongation at break (T) of the NDM and multilayered NDM (*n* = 3). All quantitative data are expressed as means ± standard deviations. *P < 0.05, **P < 0.01, ***P < 0.001 (one-way analysis of variance followed by Tukey’s multiple comparison). HA: Hyaluronic acid; IGF: insulin-like growth factor; MH: minocycline hydrochloride; MNDM: multilayered nanofiber dural mater; NDM: nanofiber dural mater without drug; PCL: polycaprolactone; TEM: transmission electron microscopy.

Three layers of the NDM were successfully produced (**Additional Figure 1**). **Figure 1A–C** illustrates the microscopic morphology of each layer of the dura mater. The fibers in each layer were randomly arranged to form an interwoven fiber network. The surface of the fibers was smooth without obvious droplets or particles. **Figure 1D** shows that no signs of delamination were found in the cross-sectional sections of the MNDM. The thickness of the MH + IGF-MNDM was approximately 180–250 μm. The fiber diameter of the PCL/HA-NDM was approximately 300–550 nm, and that of the PCL-NDM was approximately 300–550 nm. The fiber diameter of the MH + IGF-NDM fell in the range of 450–700 nm (**Additional Figure 2**).

**Figure 1E–H** shows that the water contact angles of the PCL/HA-NDM and MH + IGF-NDM groups were 0°, demonstrating good hydrophilicity, whereas the water contact angle of the PCL-NDM group was 139.97° ± 0.79°, indicating excellent hydrophobicity. The water contact angle of the MH + IGF-MNDM group was 0° because water droplets were added from the inner dural direction.

In this study, we prepared nanofibers with a dual-core-shell structure by combining emulsion electrospinning and coaxial electrospinning technologies. We verified the core-shell structure of emulsion nanofibers using fluorescence technology. The merged fluorescence micrograph showed that the aqueous phase of the emulsion nanofibers was successfully loaded into the core layer of the emulsion nanofibers (**Figure 1I–K**). Therefore, we sought to further verify the dual-core-shell structure of the nanofibers. The fluorescence microscopy results showed that the emulsion nanofibers were well encapsulated in the shell nanofibers (**Figure 1L–N**). Taken together, these results demonstrate the successful construction of nanofibers with a dual-core-shell structure. Furthermore, using transmission electron microscopy, we showed that the nanofibers have a good core-shell structure (**Figure 1O**).

To study the *in vitro* release behavior of the NDM, we plotted the cumulative percentage release curve. There was a substantial release of MH during the early stage. The cumulative release of MH within 1 hour reached 51.81% ± 2.08%, and from 1 to 12 hours, the cumulative release of MH increased to 68.32% ± 2.4%. From days 1 to 3, the cumulative release of MH increased from 70.12% ± 0.97% to 76.59% ± 0.46%. The release curve gradually plateaued (**Figure 1P**). We used RB as the drug model of the core layer of the dura mater for the release experiment. The release profile of RB is characterized by a long-term continuous release, with a duration of up to 42 days. The cumulative release of RB increased from 29.2% ± 6.7% to 44.91% ± 8% from days 1 to 5. The early large-scale release contributed to the repair of the damaged nerves. The cumulative release of RB gradually increased to 63.6% ± 5.8% from days 5 to 14 and 94.2% ± 2.9% from days 14 to 42. The release of RB continued before gradually stabilizing (**Figure 1Q**).

The dura mater must have considerable strength to protect the brain from external pressure and provide an optimal environment for nerve repair (Wang et al., 2021). We found that the tensile strength of the MH + IGF-MNDM was 9.88 ± 0.6 MPa, and the maximum strain was 53.99% ± 6.5%. The tensile strength of the MH + IGF-NDM was 10.27 ± 0.73 MPa, and the maximum strain was 27.95% ± 3.6% (**Figure 1R–T**). The tensile strength of the normal human dura mater was 7.2 ± 0.4 MPa (Khurana et al., 2024), which was considerably lower than that of the dura mater of each group. In addition, the MH + IGF-MNDM group did not show a significant difference in tensile strength from the MH + IGF-NDM group, although its maximum strain was higher. When we introduced the PCL/HA-NDM and PCL-NDM, MH + IGF-MNDM, high tensile strength was maintained; moreover, the flexibility of the material was enhanced, which provided brain tissue with more effective protection and support.

The pH value of MH + IGF-MNDM was maintained at a range of 7.25–7.5 at the time points of 10, 20, 30, 60, 90, 120, 150, and 180 days (**Additional Figure 3A**). The MH + IGF-MNDM showed sustained and slight mass loss at the different time points of 10, 20, 30, 60, 90, 120, 150, and 180 days. The dura mater was only 84% ± 2% of its initial mass at 180 days of degradation (**Additional Figure 3B**). We examined the fiber morphology on the surface and back of the MH + IGF-MNDM after 30, 60, and 90 days of degradation using scanning electron microscopy (**Additional Figure 3C**). The MH + IGF-NDM and PCL/HA-NDM showed excellent hydrophilicity, and their fiber structures became thicker during degradation. The fiber structures of the MH + IGF-NDM and PCL/HA-NDM did not break at 30, 60, or 90 days after degradation, and this strong supportive property was maintained. Therefore, this indicates that the MH + IGF-MNDM has a stable microstructure and can provide sufficient long-term support throughout the degradation process to facilitate dural recovery.

### Construction of the nerve injury model

**Figure 2:**
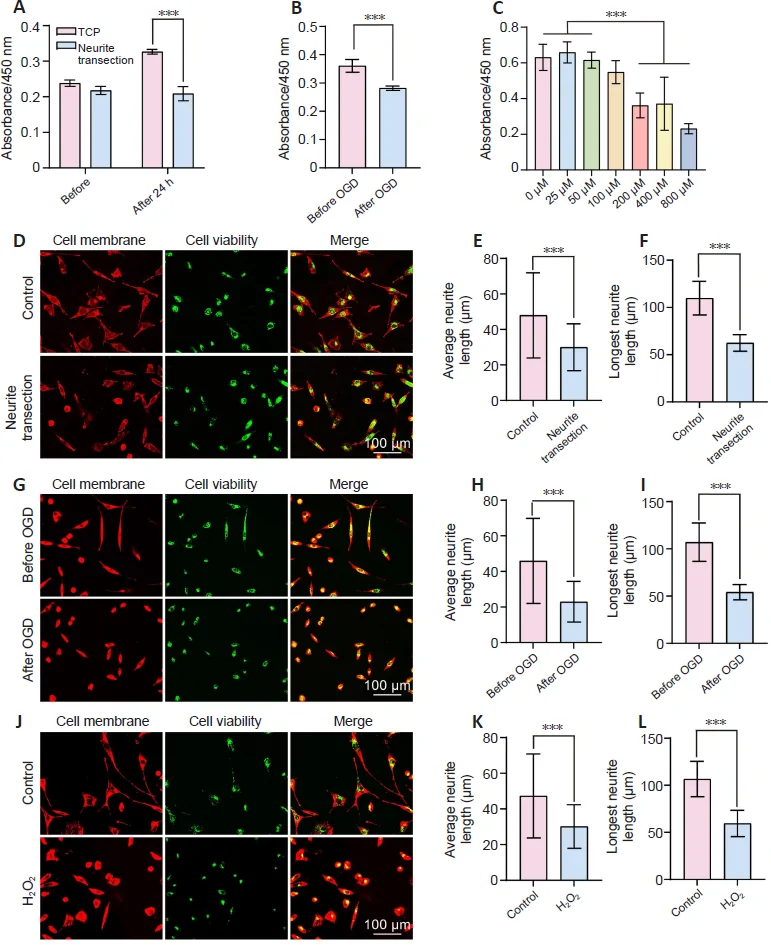
Construction of the nerve injury models. (A–C) Effects on nerve cell viability following treatment in various nerve injury models. (A) SH-SY5Y cell viability 1 day after neurite transection treatment. (B) SH-SY5Y cell viability before and after OGD treatment. (C) SH-SY5Y cell viability 1 day after treatment with different concentrations of H_2_O_2_. Cell viability was significantly decreased after different nerve injury interventions. (D–F) Effects of neurite transection on SH-SY5Y cells 1 day after treatment. (D) Immunofluorescence images. The neurite of SH-SY5Y cells treated with neurite transection was significantly shortened. (E) Graph of average neurite length. (F) Graph of longest neurite length. (G–I) SH-SY5Y cells before and after OGD treatment. (G) Immunofluorescence images. The neurite of SH-SY5Y treated with OGD was significantly shortened. (H) Graph of average neurite length. (I) Graph of longest neurite length. (J–L) Effects of oxidative stress on SH-SY5Y cells 1 day after treatment. (J) Immunofluorescence images. The neurite of SH-SY5Y treated with oxidative stress was significantly shortened. Scale bars: 100 μm (D, G, and J). (K) Graph of average neurite length. (L) Graph of longest neurite length. All quantitative data are expressed as means ± standard deviations (*n* = 3). ***P < 0.001 (two independent samples *t*-test used for A, B, E, F, H, I, K, L; one-way analysis of variance followed by Tukey’s multiple comparison used for C). H_2_O_2_: Hydrogen peroxide; OGD: oxygen and glucose deprivation; TCP: tricalcium phosphate.

SH-SY5Y cells were used to construct a neurite transection model to simulate primary nerve injury after TBI (Morrison et al., 2011). Cell viability decreased significantly after neurite transection treatment (*P* < 0.001; **Figure 2A**). SH-SY5Y cells were also used to construct an OGD model to simulate secondary nerve injury (Yuan et al., 2023; Liu et al., 2024), and the cells showed significant damage after OGD treatment (*P* < 0.001; **Figure 2B**). Excessive oxidative stress has been confirmed to significantly hinder nerve repair and regeneration (Islam et al., 2022; Tie et al., 2022; Zhang et al., 2023a). Thus, SH-SY5Y cells were used to construct an oxidative stress injury model to simulate secondary nerve injury. **Figure 2C** shows that SH-SY5Y cell viability decreased significantly when the concentration of H_2_O_2_ surpassed 200 μM (*P* < 0.001).

**Figure 2D** shows the significant cutting off on the neurites of neural cells. The average and longest neurite lengths in the transected group were significantly lower than those in the control group (*P* < 0.001; **Figure 2E** and **F**). The constructed neurite transection model effectively severed the neurites, which resulted in a significant reduction in neurite length and viability of neural cells. **Figure 2G** shows that both the average and longest neurite lengths of the OGD-treated group were significantly shorter than those of the control group, which demonstrated that the OGD model was successfully constructed (*P* < 0.001, **Figure 2H** and **I**). SH-SY5Y cells exposed to an H_2_O_2_ concentration exceeding 200 μM exhibited cell morphology alterations (**Additional Figure 4**). Therefore, we used an H_2_O_2_ concentration of 200 μM to induce SH-SY5Y cells, after which the average and longest neurite lengths reduced significantly (*P* < 0.001, **Figure 2J–L**). These results demonstrate that we successfully constructed the oxidative stress injury model.

### Nanofibrous dura mater promotes the survival of damaged nerves and the regeneration of neurite extension

**Figure 3:**
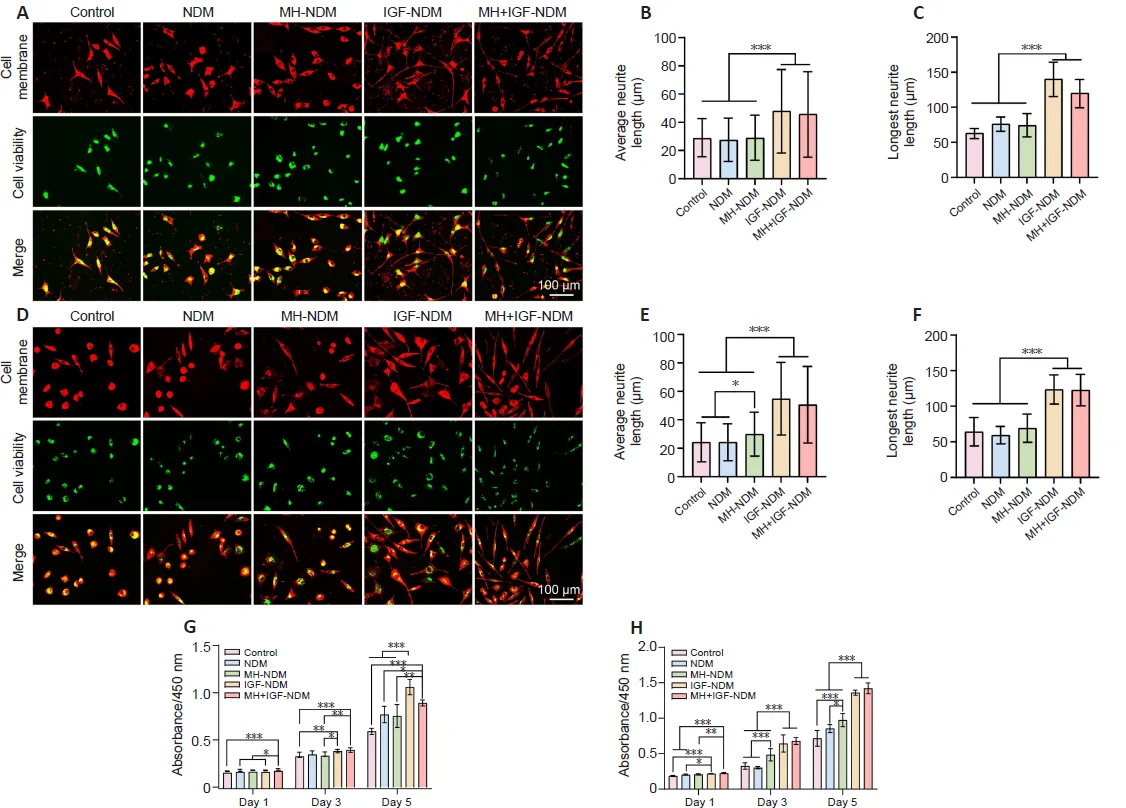
Effects of different dura mater on the neurite transection injury and oxidative stress injury models. (A–C) Effects of the MH + IGF-NDM on SH-SY5Y cells 3 days after neurite transection injury. (A) Immunofluorescence images. The MH + IGF-NDM and IGF-NDM performed better than the other groups for promoting neurite extension of SH-SY5Y cells with neurite transection injury. (B) Graph of average neurite length. (C) Graph of longest neurite length. (D–F) Effects of the MH + IGF-NDM on SH-SY5Y cells 3 days after oxidative stress injury. (D) Immunofluorescence images. The MH + IGF-NDM and IGF-NDM performed better than the other groups for promoting neurite extension of SH-SY5Y cells with oxidative stress injury. Scale bars: 100 μm (A and D). (E) Graph of average neurite length. (F) Graph of longest neurite length. (G and H) Effects of the MH + IGF-NDM on SH-SY5Y cell viability at 1, 3, and 5 days after nerve injury. (G) Neurite transection injury. (H) Oxidative stress injury. All quantitative data are expressed as means ± standard deviations (*n* = 3). *P < 0.05, **P < 0.01, ***P < 0.001 (one-way analysis of variance followed by Tukey’s multiple comparison). IGF: Insulin-like growth factor; MH: minocycline hydrochloride; NDM: nanofiber dural mater without drug.

IGF-1 plays a variety of physiological roles in the human body. It has an important protective effect on neural cells and is effective in restoring damaged nerve function (Liu et al., 2025). Therefore, we further examined the reparative effects of the dura mater on different nerve injury models after TBI via long-term sustained release of IGF-1. The effect of the MH + IGF-NDM on primary nerve injury after TBI was studied by using the neurite transection model. After 3 days of different dural interventions, the average and longest neurite lengths of cells in the MH + IGF-NDM and IGF-NDM groups were significantly longer than those in the other groups (*P* < 0.001; **Figure 3A–C**). Cell viability of the MH + IGF-NDM and IGF-NDM groups was higher than that of the other groups by day 5 post-injury. Therefore, the MH + IGF-NDM promoted cell survival and proliferation after neurite transection (**Figure 3G**). The MH + IGF-NDM significantly enhanced cell proliferation and neurite extension after neurite transection via sustained IGF-1 release, which suggests that this NDM facilitates recovery from injury during the acute phase.

In regard to the effects of the MH + IGF-NDM on nerve injury caused by oxidative stress in the acute phase after TBI, **Figure 3D–F** shows that the average and longest neurite lengths of cells in the MH + IGF-NDM and IGF-NDM groups were significantly longer than those in the other groups (*P* < 0.001). **Figure 3H** shows that cell viability of the MH + IGF-NDM and IGF-NDM groups was higher than that of the other groups at each time point following oxidative stress injury. Therefore, the MH + IGF-NDM can effectively repair oxidative stress injury after TBI by releasing IGF-1.

**Figure 4:**
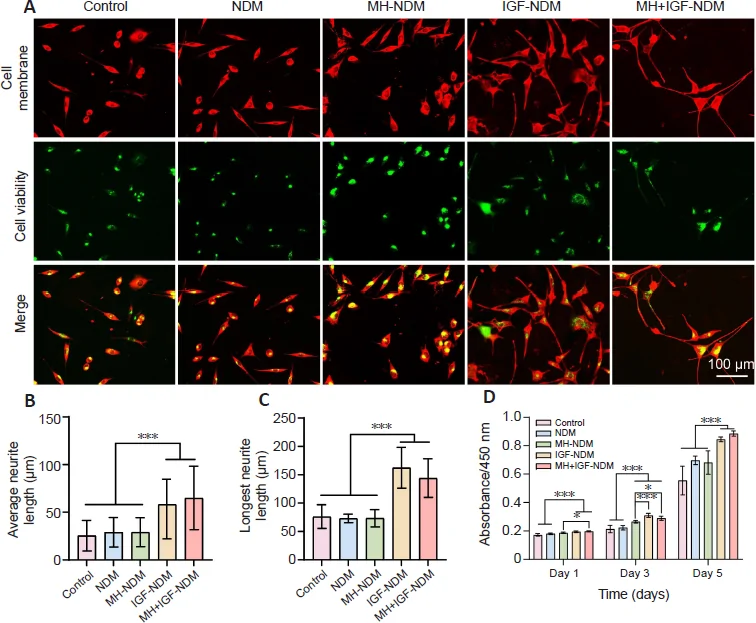
Effects of different dura mater on the oxidative stress injury model. (A–C) Effects of the different dura mater on SH-SY5Y cells 3 days after oxidative stress injury. (A) Immunofluorescence images. The MH + IGF-NDM and IGF-NDM performed better than the other groups for promoting neurite extension of SH-SY5Y cells with oxidative stress injury. Scale bar: 100 μm (A). (B) Graph of average neurite length. (C) Graph of longest neurite length. (D) Effects of the different dura mater on SH-SY5Y cell viability 1, 3, and 5 days after oxidative stress injury. All quantitative data are expressed as means ± standard deviations (*n* = 3). *P < 0.05, ***P < 0.001 (one-way analysis of variance, followed by Tukey’s multiple comparison). IGF: Insulin-like growth factor; MH: minocycline hydrochloride; NDM: nanofiber dural mater without drug.

**Figure 5:**
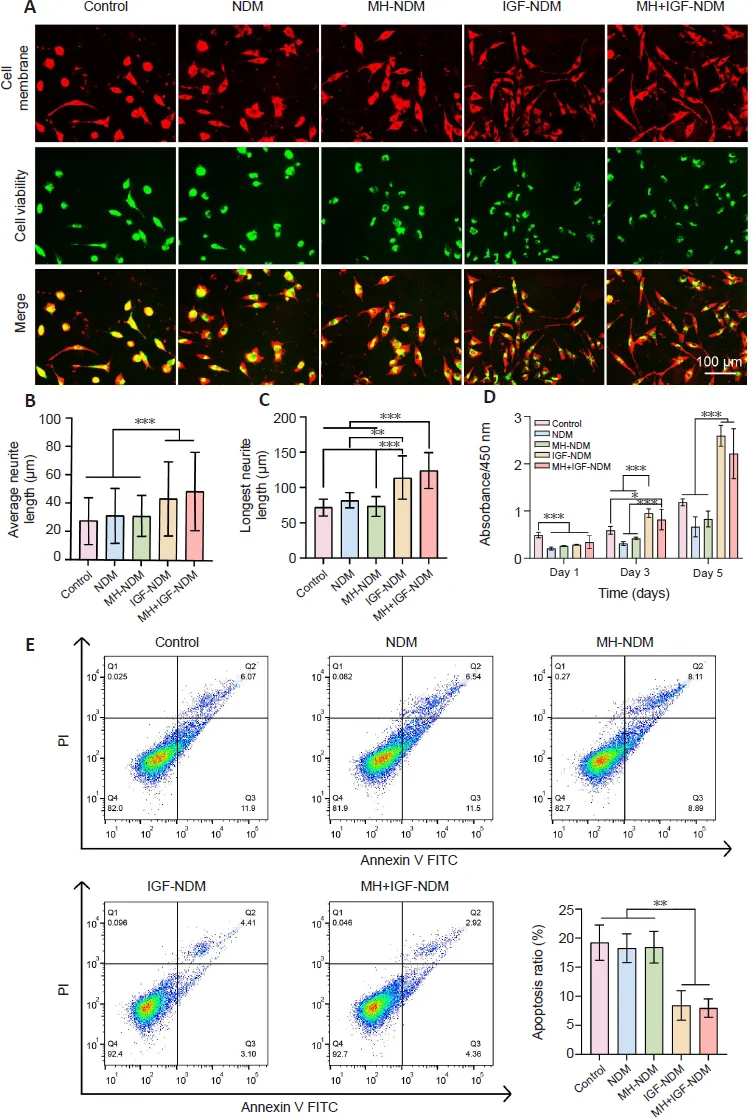
Effects of the different dura mater on the OGD injury model. (A–C) Effect of the different dura mater on SH-SY5Y cells 3 days after OGD injury. (A) Immunofluorescence images. The MH + IGF-NDM and IGF-NDM performed better than the other groups for promoting neurite extension of SH-SY5Y cells with OGD injury. Scale bar: 100 μm (A). (B) Graph of average neurite length. (C) Graph of longest neurite length (*n* = 3). (D) Effects of the MH + IGF-NDM on SH-SY5Y cell viability 1, 3, and 5 days after OGD injury (*n* = 3). (E) Apoptosis of SH-SY5Y cells 3 days after OGD injury for different dura mater (*n* = 3). All quantitative data are expressed as means ± standard deviations. *P < 0.05, **P < 0.01, ***P < 0.001 (one-way analysis of variance followed by Tukey’s multiple comparison). FITC: Fluorescein isothiocyanate; IGF: insulin-like growth factor; MH: minocycline hydrochloride; NDM: nanofiber dural mater without drug; OGD: oxygen and glucose deprivation.

Nerve repair following TBI is a prolonged process. Secondary nerve injury often damages nerve cells, which can affect a wide range of body functions, such as sensory disorders, motor disorders and cognitive disorders (Sherman et al., 2016; Akamatsu and Hanafy, 2020). We used OGD and oxidative stress injury models to simulate secondary neurological injury that occurs during the subacute phase after TBI. **Figure 4A–C** shows that the mean and longest neurite lengths were significantly longer in the MH + IGF-NDM and IGF-NDM groups than in the other groups (*P* < 0.001). Furthermore, **Figure 4D** shows that cell viability was significantly higher in the MH + IGF-NDM and IGF-NDM groups than in the other groups (*P* < 0.001). The MH + IGF-NDM supported the survival of damaged cells via the sustained release of IGF-1, which aided the recovery of damaged nerves. **Figure 5A–C** shows that the average and longest neurite lengths of cells in the MH + IGF-NDM and IGF-NDM groups were significantly longer than those in the other groups (*P* < 0.01). Additionally, **Figure 5D** shows that cell viability was significantly higher in the MH + IGF-NDM and IGF-NDM groups than in the other groups at days 3 and 5 post-injury. The MH + IGF-NDM enhanced cell survival and neurite extension after OGD and promoted the recovery of damaged nerves via long-term sustained release of IGF-1. **Figure 5E** shows that the apoptosis rate of SH-SY5Y cells of the MH + IGF-NDM and IGF-NDM groups was significantly lower than that of the other groups (*P* < 0.01). These results suggest that the MH + IGF-NDM promoted significant nerve repair and protective effects via the continuous delivery of IGF-1 and that the MH + IGF-NDM can effectively release IGF-1 after TBI. Therefore, this NDM may play a beneficial role in nerve repair at different stages of various nerve injury models and contribute to the repair of damaged nerves following TBI.

### Nanofibrous dura mater promotes immunomodulation and neural survival

**Figure 6:**
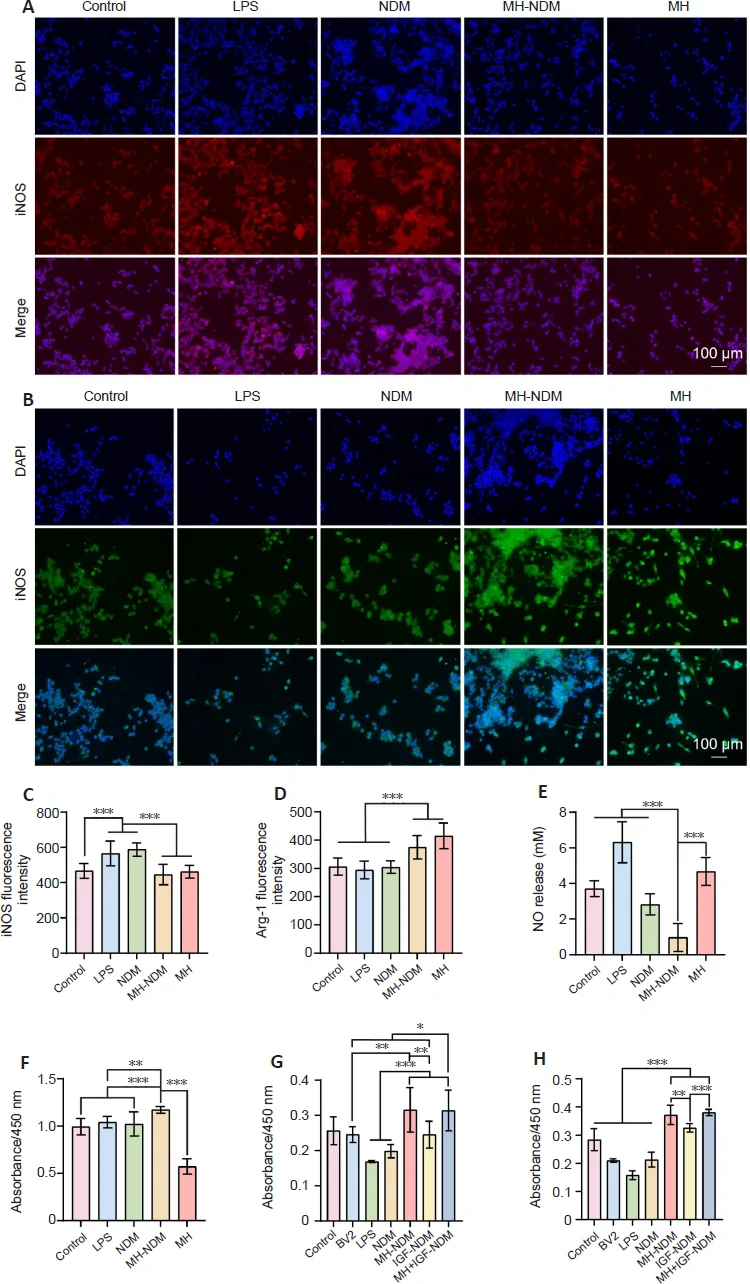
The immunomodulatory properties of the MNDM and their impact on nerve cell survival. (A–D) Effect of the MH + IGF-NDM on the polarization phenotype of BV2 cells. Immunofluorescence images of iNOS (red, Alexa Fluor 594) (A) and Arg-1 (green, Alexa Fluor 488) (B). Scale bars: 100 μm (A and B). Fluorescence intensity of iNOS (C) and Arg-1 (D). (E) Effect of the MH + IGF-NDM on NO release from BV2 cells after LPS treatment. (F) Effect of the MH + IGF-NDM on the viability of BV2 cells following LPS treatment. (G and H) Effect of conditioned medium modulation of BV2 cells on SH-SY5Y cell viability 3 days after neurite transection injury (G) and OGD injury (H). All quantitative data are expressed as means ± standard deviations (*n* = 3). *P < 0.05, **P < 0.01, ***P < 0.001 (one-way analysis of variance followed by Tukey’s multiple comparison). Arg-1: Arginase-1; DAPI: 4′,6-diamidino-2-phenylindole; IGF: insulin-like growth factor; iNOS: inducible nitric oxide synthase; LPS: lipopolysaccharide; MH: minocycline hydrochloride; NDM: nanofiber dural mater without drug; NO: nitric oxide; OGD: oxygen and glucose deprivation.

TBI faces a complex microenvironment. Appropriate medical interventions can prevent further damage and improve long-term outcomes (He et al., 2020; Chen et al., 2022; Estrella et al., 2025). We investigated whether the dura mater can reduce an excessive inflammatory response *in vitro* using the LPS-induced BV2 cell inflammation model. INOS is a common marker for the M1 type of BV2 cells, and arginase-1 is a common marker for the M2 type of BV2 cells (Khoshnavay Foumani et al., 2024; Li et al., 2025). **Figure 6A–D** shows that the expression level of iNOS was significantly lower in the MH-NDM and MH groups than in the LPS and NDM groups (*P* < 0.001), whereas the arginase-1 expression level was significantly higher in the MH-NDM and MH groups than in the control, LPS, and NDM groups (*P* < 0.001). Additionally, the level of NO release was significantly lower in the MH-NDM group than in the other groups (*P* < 0.001; **Figure 6E**). These results indicated that the MH-NDM successfully polarized the BV2 cells from a pro-inflammatory M1 phenotype to an anti-inflammatory M2 phenotype. **Figure 6F** shows that cell viability was significantly higher in the MH-NDM group than in the other groups (*P* < 0.01). In contrast, cell viability of the MH group was significantly lower than that of the other groups, which may be related to the potential toxicity of a large amount of MH being released into the cells over a short period (*P* < 0.001). By embedding MH in the dura mater, the sustained release mechanism of the dura mater can effectively reduce the cytotoxic damage of cultured neural cells. We investigated the role of the MH + IGF-NDM in injured nerve repair by modulating the polarization of BV2 cells. In both the neurite transection and OGD injury models, cell viability was lower in the LPS group than in the other groups (**Figure 6G** and **H**). LPS may induce the polarization of BV2 cells to the M1 type, increase the production of inflammatory factors, and inhibit the repair of damaged neural cells. The cell viability of the MH + IGF-NDM and MH-NDM groups was significantly higher than that of other groups (*P* < 0.05), which may be because MH regulates the polarization of BV2 cells from a pro-inflammatory M1 phenotype to an anti-inflammatory M2 phenotype, reducing the secretion of excessive inflammatory factors and promoting the survival and repair of damaged neural cells. Therefore, the MH + IGF-NDM has a beneficial anti-inflammatory effect on nerve injury and reduces damage to neural cells due to excessive inflammatory factors.

### Nanofibrous dura mater exhibits excellent antibacterial properties

The antibacterial rate of the dura mater against *S. aureus* and *E. coli* was compared with that of the control group. As shown in **Additional Figure 5**, the antibacterial rate against *S. aureus* was significantly higher in the MH + IGF-NDM and MH-NDM groups than in the IGF-NDM and NDM groups (*P* < 0.001). The MH + IGF-NDM effectively eliminated *S. aureus*. **Additional Figure 6** shows that both the MH + IGF-NDM and MH-NDM groups effectively inhibited the growth of and eliminated *E. coli*. Furthermore, the antibacterial effects of the MH + IGF-NDM and MH-NDM groups against *E. coli* were significantly greater than those of the IGF-NDM and NDM groups (*P* < 0.001). Therefore, the MH + IGF-NDM has excellent antibacterial properties and can effectively kill *S. aureus* and *E. coli*, which will reduce the risk of neurosurgical surgical site infection and improve postsurgical prognosis.

### Nanofibrous dura mater possesses good anti-adhesion properties

**Figure 7:**
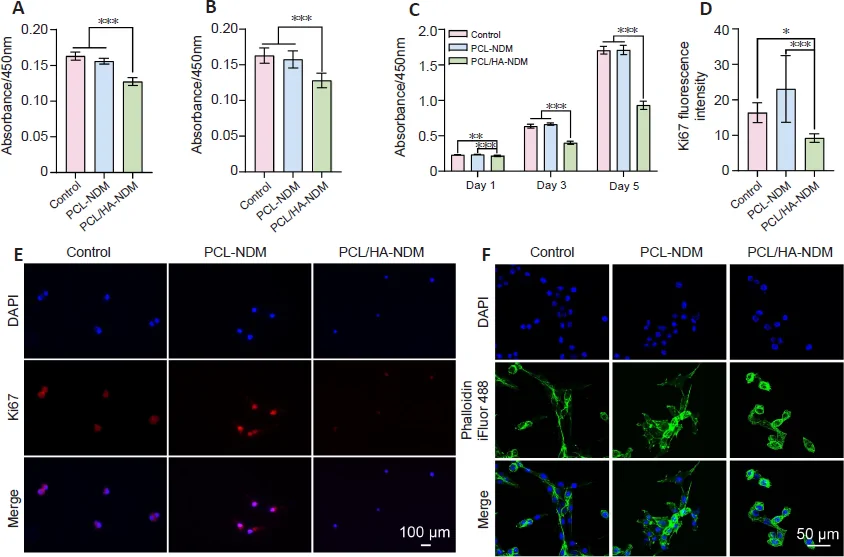
Anti-adhesive capacity of the MNDM *in vitro*. (A and B) Cell viability of NIH3T3 cells on the PCL/HA-NDM 6 (A) and 12 hours (B) after treatment. (C) Cell viability of NIH3T3 cells cultured on the PCL/HA-NDM for 1, 3, and 5 days. (D and E) Immunofluorescence images showing the proliferation of NIH3T3 cells cultured on the control, PCL-NDM, and PCL/HA-NDM for 3 days. (D) Ki67 fluorescence intensity. All quantitative data are expressed as means ± standard deviations (*n* = 3). *P < 0.05, **P < 0.01, ***P < 0.001 (one-way analysis of variance, followed by Tukey’s multiple comparison). (E) Immunofluorescence images of Ki67 (red, Alexa Fluor 594). (F) Immunofluorescence images showing the growth morphology of NIH3T3 cells cultured on the control, PCL-NDM, and PCL/HA-NDM for 3 days. The synaptic length of the PCL/HA-NDM group was shorter than that of the other groups. Scale bars: 100 μm (E) and 50 μm (F). DAPI: 4′,6-Diamidino-2-phenylindole; HA: hyaluronic acid; NDM: nanofiber dural mater without drug; PCL: polycaprolactone.

During wound healing, fibroblast activation enhances ECM secretion and promotes wound healing and tissue regeneration. However, excessive ECM can lead to adhesion, fibrosis, and scar formation, which hinder repair and regeneration (Cheng et al., 2019; Talbott et al., 2022). The CCK-8 was used to determine the initial number of cells in each group, 6 and 12 hours after seeding. **Figure 7A** and **B** shows that the initial cell number was significantly lower in the PCL/HA-NDM group than in the other groups in all cases (*P* < 0.001). The addition of HA reduced the ability of NIH3T3 cells to adhere to the dura mater. **Figure 7C** shows a gradual increase in cell viability from days 1 to 5 across all groups. However, the cell viability of the PCL/HA-NDM group was significantly lower than that of the control and PCL-NDM groups (*P* < 0.01). Subsequently, we used Ki67-labeled fibroblasts to analyze the effect of the PCL/HA-NDM on cell proliferation ability. **Figure 7D** and **E** show that Ki67 expression was significantly lower in the PCL/HA-NDM group than in the other groups (*P* < 0.05). The PCL/HA-NDM inhibited fibroblast viability by inhibiting excessive fibroblast proliferation. **Figure 7F** shows that the length of synapses of the PCL/HA-NDM group was shorter than that of the other groups, and the number of cells in the visual field of the PCL/HA-NDM group was smaller than that of the other groups. Taken together, our findings suggest that the PCL/HA-NDM plays an anti-adhesive role by inhibiting the excessive proliferation of fibroblasts and reducing the ability of fibroblasts to adhere to the membrane.

### Nanofibrous dura mater shows good barrier performance

**Figure 8:**
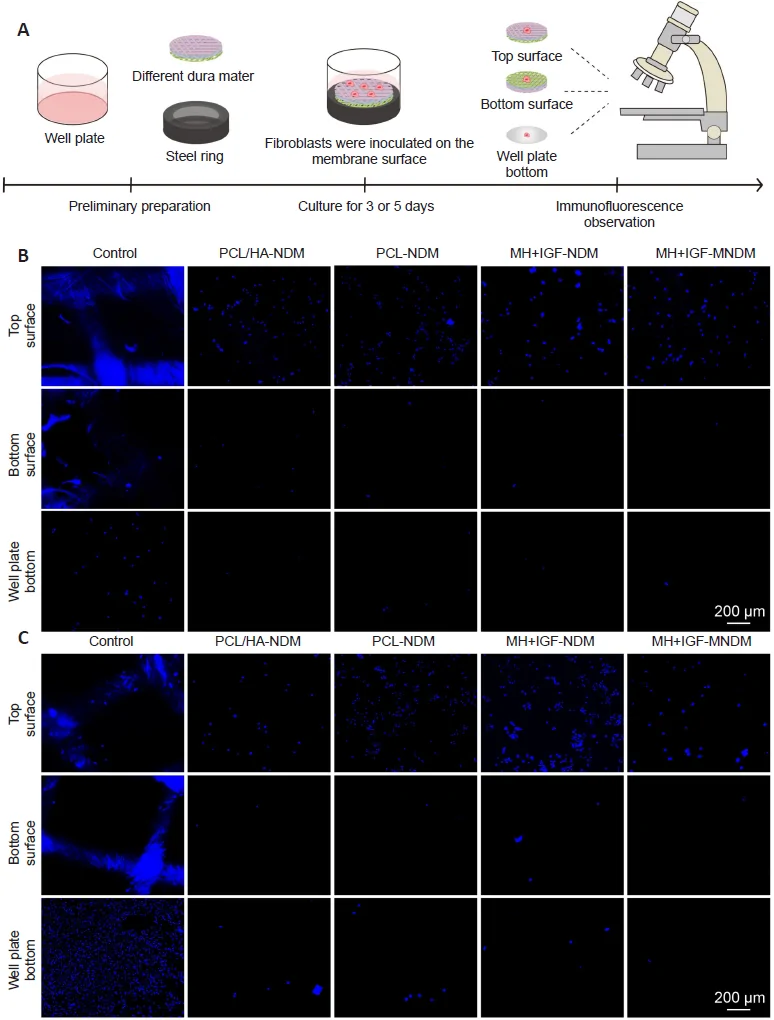
Barrier capacity of MNDM *in vitro*. (A) Schematic representation of the barrier experiments (created with Adobe Illustrator 2017). (B and C) Immunofluorescence images illustrating the penetration of fibroblasts cultured on the PCL/HA-NDM, PCL-NDM, MH + IGF-NDM, MH + IGF-MNDM, and control for 3 (B) and 5 (C) days. All groups effectively blocked the penetration of NIH3T3 cells into the bottom of the well plate on days 3 and 5 of the barrier experiment. Scale bars: 200 μm (B and C). HA: Hyaluronic acid; MH: minocycline hydrochloride; MNDM: multilayered nanofiber dural mater; NDM: nanofiber dural mater without drug; PCL: polycaprolactone.

We confirmed the barrier capacity by seeding NIH3T3 cells into steel rings with various dura mater (**Figure 8A**). **Figure 8B** and **C** show that cells in the control group easily crossed the upper surface of the membrane to reach the bottom of the well plate. However, cells on the top surface of the dura mater proliferated over time in all groups, and the number of cells at the bottom of the well plate was significantly lower in all groups than in the control group, particularly in the MH + IGF-MNDM group (*P* < 0.001; **Additional Figure 7**). The dense structure of the dura mater effectively prevented the penetration of fibroblasts from the upper surface to the lower surface and ultimately to the bottom of the culture plate. Therefore, the MH + IGF-MNDM showed good barrier performance.

### Nanofibrous dura mater has excellent anti-leakage properties

**Figure 9:**
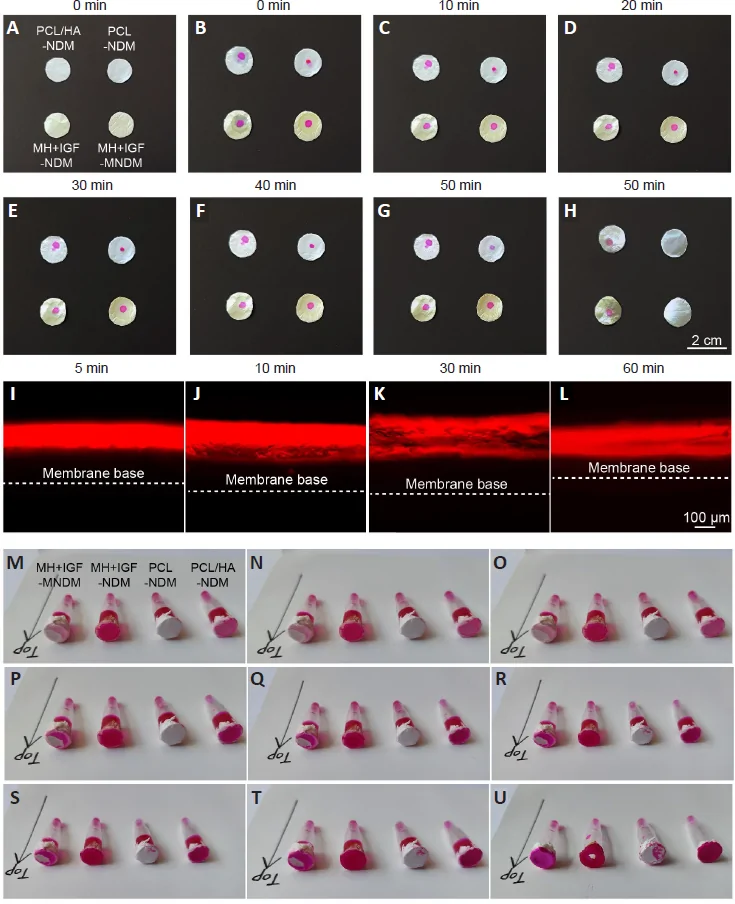
Anti-leakage properties of the MNDM *in vitro*. (A–H) Pressure-free droplet penetration experiments. (A) 0 min (without RB solution). The frontal side of the dura mater after 0 (B), 10 (C), 20 (D), 30 (E), 40 (F), and 50 minutes (G), and the reverse side of the dura mater after 50 minutes (H) after the drop of RB solution. No red liquid droplets penetrated to the bottom of the dura mater in the MH + IGF-MNDM or PCL-NDM group. However, penetration occurred in the other two groups. (I–L) Fluorescent droplet permeation experiments of the MNDM at 5 (I), 10 (J), 30 (K), and 60 minutes (L). No red droplets permeated through the MH + IGF-MNDM at any time point. The dashed lines indicate the lower surface of the dura mater. Scale bars: 2 cm (A–H), and 100 μm (I–L). (M–U) Cross sections at different time points in the inverted centrifugal tube penetration experiment. Pictures of dura mater cross sections after 5 (M), 10 (N), 20 (O), 30 (P), 60 (Q), and 90 minutes (R), and 2 (S), 3 (T), and 20 hours (U). The surface of the dura mater showed a trend of gradually deeper red staining over time. The MH + IGF-MNDM and PCL-NDM groups were consistently less pigmented than the other two groups. Surface holes were only observed in the MH + IGF-NDM group after 20 hours. IGF: Insulin-like growth factor; MH: minocycline hydrochloride; MNDM: multilayered nanofiber dural mater; NDM: nanofiber dural mater without drug; PCL: polycaprolactone; RB: rhodamine B.

Dural defects resulting from brain injury may lead to CSF leakage, which may impact the process of nerve repair (Liao et al., 2021; Zhu et al., 2022). To examine the anti-leakage properties of the NDM, we performed pressure-free droplet permeation experiments. The dura mater in each group was cut into 14-mm-diameter circles and fixed onto a table (**Figure 9A**). An RB solution was dripped onto the meninges of each group (**Figure 9B**). The MH + IGF-NDM and PCL/HA-NDM groups exhibited immediate spreading, whereas RB solution was added from the inner layer direction of the MH + IGF-MDNM group; a similar phenomenon was observed in the MH + IGF-NDM group. The RB solution forms droplets on the surface of the PCL-NDM owing to its hydrophobicity. At 10, 20, 30, and 40 minutes after application of the RB solution, the surface solutions of the MH + IGF-NDM, PCL/HA-NDM, and MH + IGF-MDNM groups were spread uniformly, whereas the surface of the PCL-NDM group retained the droplet shapes (**Figure 9C–F**). At 50 minutes, the red droplets on the surface of the PCL-NDM group exhibited a lighter hue and reduced volume (**Figure 9G**). The efficacy of each group in preventing leakage was also evaluated by monitoring the color change on the lower surface of the membrane. At 50 minutes, no red droplet was observed on the lower membrane surface in the PCL-NDM or MH + IGF-MDNM groups. In contrast, a clear red droplet was evident on the lower surface membrane of the MH + IGF-NDM and PCL/HA-NDM groups (**Figure 9H**). Therefore, the PCL-NDM and MH + IGF-MDNM demonstrated a better anti-leakage effect than the MH + IGF-NDM and PCL/HA-NDM. Given these findings, we further evaluated the MH + IGF-MDNM using fluorescent dye penetration experiments and found that red fluorescence could not penetrate the lower surface of the MH + IGF-MDNM at any time point (i.e., at 5, 10, 30, or 60 minutes of applying 10 μL of RB solution; **Figure 9I–L** and **Additional Figure 8**). Our results indicated that, consistent with previous findings, the MH + IGF-MDNM fused with the PCL-NDM offers effective anti-leakage properties and thus favorable conditions for nerve repair.

To further evaluate leakage prevention performance, we observe the static leakage prevention ability of different dura mater against 1 mL of RB solution under the effect of gravity (**Additional Figure 9A** and **B**). **Figure 9M–T** show that the solutions rapidly permeated the membrane at the orifice in the MH + IGF-NDM and PCL/HA-NDM groups, although the solutions did not leak. In the MH + IGF-MDNM group, red maceration was also noted. However, the degree of red maceration at the orifice membrane was markedly lower in the MH + IGF-MNDM and PCL-NDM groups than in the other groups. The PCL-NDM group showed strong blocking ability, and the red droplets failed to penetrate through the dura mater. Over time, the red impregnation of each group increased gradually, and the PCL-NDM group consistently showed significantly lower red impregnation than the other groups. After 20 hours, holes were observed in the MH + IGF-NDM section where red droplets remained. No red droplets flowed down the membrane surface in any of the other groups (**Figure 9U** and **Additional Figure 9C**). In addition, the MH + IGF-MNDM, PCL-NDM, and PCL/HA-NDM groups all showed good static leakage protection.

To further investigate the anti-leakage ability of different dura mater under dynamic aqueous solutions. None of the groups leaked water, which indicated that the prepared dura mater of each group had good anti-leakage ability for short-term dynamic aqueous solutions (**Additional Figure 10**). Taken together, our results showed that the MH + IGF-MNDM has relatively excellent anti-leakage performance under both dynamic and static solution conditions.

### Nanofibrous dura mater exhibits excellent biocompatibility

#### In vitro biocompatibility evaluation

**Figure 10:**
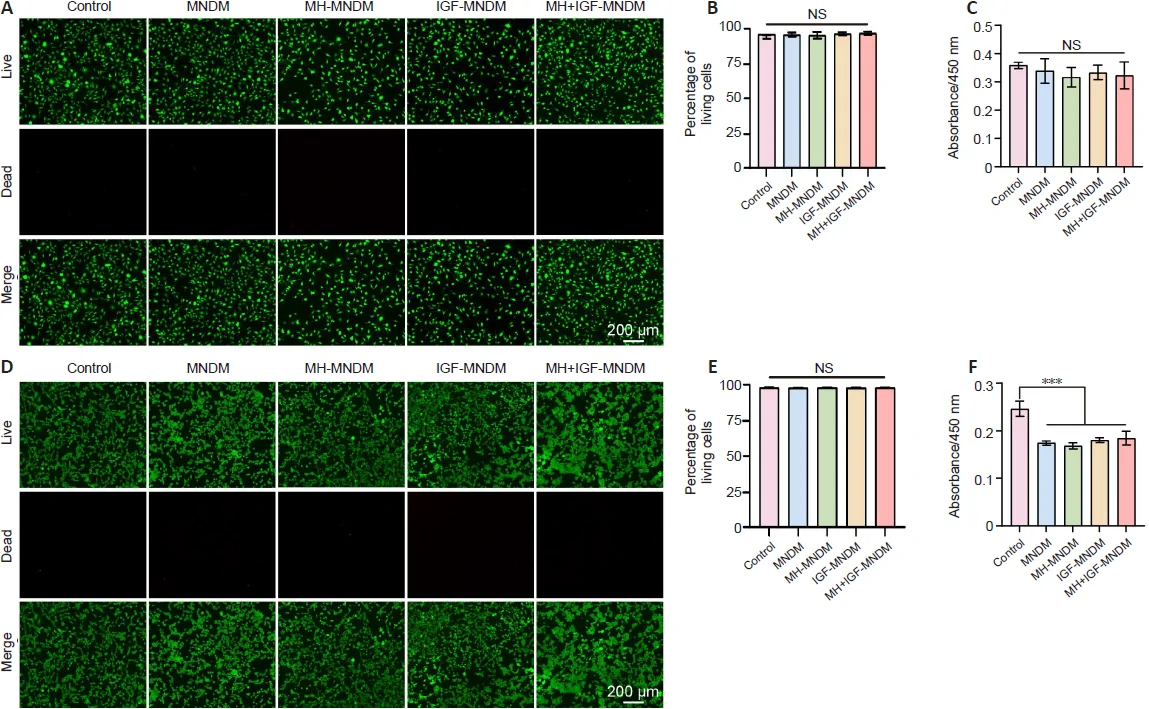
*In vitro* biocompatibility properties of the MNDM. (A–C) The effect of the MNDM on the biocompatibility of SH-SY5Y cells. (A, B) Live-dead staining (A) and graph (B) showing the effects of the MH + IGF-MNDM on SH-SY5Y cells after 1 day. Different groups of dura mater have good biocompatibility with SH-SY5Y cells. (C) Cell viability of SH-SY5Y cells following 3 days of the MH + IGF-MNDM intervention. (D–F) The impact of the MNDM on NIH3T3 cell biocompatibility. Live-dead staining (D) and graph (E) illustrating the impact of the MH + IGF-MNDM on NIH3T3 cells after 1 day. Different dura mater groups had good biocompatibility with NIH3T3 cells. Scale bars: 200 μm (A and D). (F) Cell viability of NIH3T3 cells after 3 days of the MH + IGF-MNDM intervention. All quantitative data are expressed as mean ± standard deviations (*n* = 3). ***P < 0.001 (one-way analysis of variance followed by Tukey’s multiple comparison). IGF: Insulin-like growth factor; MH: minocycline hydrochloride; MNDM: multilayered nanofiber dural mater; NS: not significant.

The cytotoxicity of the MNDM was evaluated using live/dead staining and the CCK-8 (Li et al., 2018; Tan et al., 2022). The live/dead staining results showed that more than 95% of SH-SY5Y cells survived in all groups, and there were no significant differences among groups (*P* > 0.05; **Figure 10A** and **B**). The CCK-8 results demonstrated that the viability of SH-SY5Y cells was not affected by the presence of a triple-layer dura mater (*P* > 0.05; **Figure 10C**). These results suggest that the MH + IGF-MNDM exhibits favorable cytocompatibility with SH-SY5Y cells.

The live/dead staining results of the NIH3T3 cells showed that more than 95% of the cells in each group survived, and there were no significant differences among groups. The MH + IGF-MNDM showed good biocompatibility (*P* > 0.05; **Figure 10D** and **E**). The CCK-8 results showed that the viability of NIH3T3 cells in the control group was significantly higher than that in the other groups (*P* < 0.001; **Figure 10F**). This may be attributed to the presence of HA in the outer layer of the PCL/HA-NDM, which inhibits the proliferation of fibroblasts. This is consistent with previous experimental findings.

#### In vivo biocompatibility

**Figure 11:**
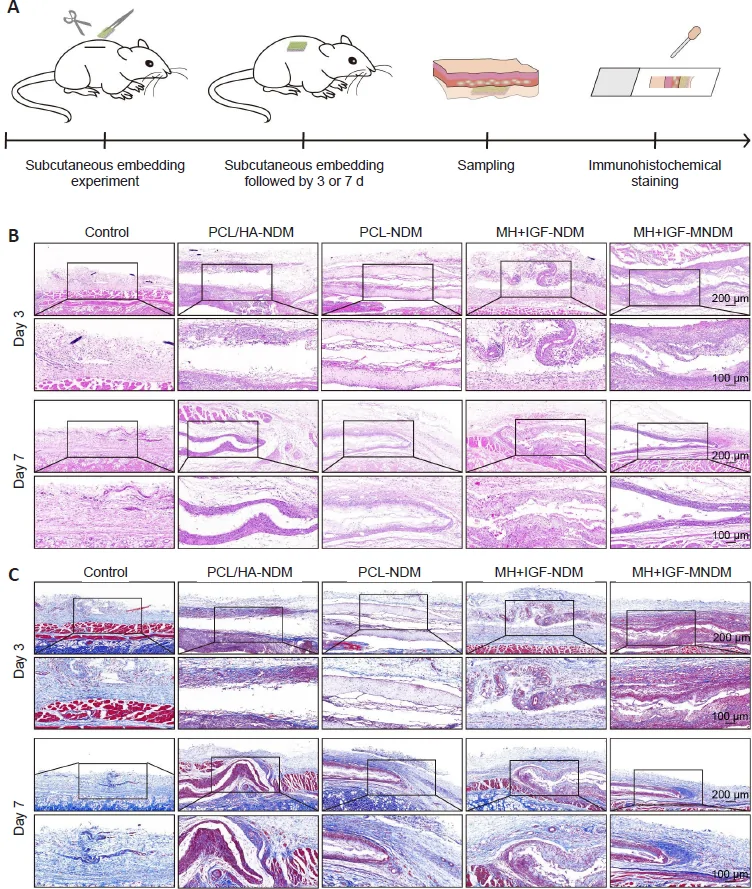
*In vivo* biocompatibility of the MNDM. (A) Schematic representation of *in vivo* biocompatibility experiments. (B, C) Hematoxylin and eosin staining (B) and Masson staining (C) of different dura mater samples, 3 and 7 days after implantation *in vivo*. Inflammation was reduced in all groups from 3 to 7 days after implantation. Collagen deposition was observed in the surrounding tissues of all groups at 3 and 7 days after implantation. Scale bars: 200 μm and 100 μm (enlarged). H&E: Hematoxylin & eosin; HA: hyaluronic acid; IGF: insulin-like growth factor; MH: minocycline hydrochloride; MNDM: multilayered nanofiber dural mater.

We investigated the *in vivo* biocompatibility of the prepared dura mater using subcutaneous embedding experiments (**Figure 11A**). All animals survived the implantation of different types of dura mater and showed no signs of infection. **Figure 11B** shows that the number of inflammatory cells was relatively higher at 3 days after implantation than at 7 days, which may be attributed to the early inflammatory response of the body to external injury. Following a 7-day implantation period, inflammation decreased across all groups. There were no severe inflammatory reactions or noticeable fibrous envelope formations. This suggests that the PCL/HA-NDM, PCL-NDM, MH + IGF-NDM, and MH + IGF-MNDM possess favorable biocompatibility. A certain degree of cellular infiltration was observed in the implanted dura mater of all groups. In particular, the MH + IGF-MNDM group exhibited a notable cell-free infiltration area. This may be related to the dense fibrous structure of the prepared dura mater, which would effectively prevent excessive cellular infiltration. The Masson staining results showed some degree of collagen deposition in the surrounding tissues at 3 and 7 days following dura mater implantation in all groups, which indicated tissue repair (**Figure 11C**). Collectively, these results suggest that the NDM of each group exhibited good biocompatibility following implantation.

### Nanofibrous dura mater induces excellent anti-inflammatory effects *in vivo*

**Figure 12:**
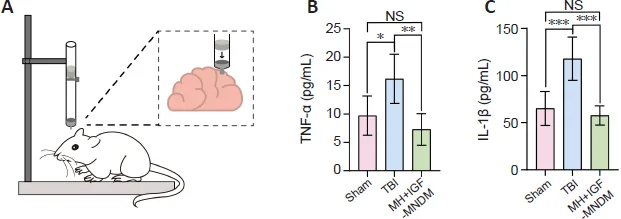
*In vivo* anti-inflammatory effects of the NDM. (A) Schematic diagram of the TBI model. (B, C) Levels of TNF-α (B) and IL-1β (C) in brain tissues of animals 3 days after TBI. There was no significant difference in the expression levels of TNF-α and IL-1β between the MH + IGF-MNDM and sham groups. All quantitative data are expressed as means ± standard deviations (*n* = 6). *P < 0.05, **P < 0.01, ***P < 0.001 (one-way analysis of variance followed by Tukey’s multiple comparison). IGF: Insulin-like growth factor; IL-1β: interleukin-1β; MH: minocycline hydrochloride; MNDM: multilayered nanofiber dural mater; NS: not significant; TBI: traumatic brain injury; TNF-α: tumor necrosis factor-α.

To investigate the regulatory role of the MH + IGF-MNDM on the acute inflammatory response following TBI, we established a rat TBI injury model using a TBI impact device (**Figure 12A**). Excessive release of proinflammatory cytokines after TBI exacerbates secondary nerve injury and inhibits the repair process (Roth et al., 2014). For example, excessive release of inflammatory factors causes acute neuronal degeneration, inhibition of neurite regeneration, and aggravation of other pathological processes (Sharma et al., 2020; Doğanyiğit et al., 2022). **Figure 12B** and **C** show that the expression levels of TNF-α and IL-1β were significantly higher in the TBI model group than in the sham group, which confirmed that TBI successfully induced an excessive inflammatory response in the acute phase (*P* < 0.05). Compared with the TBI group, the TBI + MH + IGF-MNDM group showed significantly lower expression levels of TNF-α and IL-1β (*P* < 0.01). The MH + IGF-MNDM effectively inhibited the severe inflammatory response induced by TBI and the expression of inflammatory factors to a slightly lower degree than the sham group (craniotomy). However, the TNF-α and IL-1β levels of the MH + IGF-MNDM group at 3 days after injury were not significantly different from those of the sham group (*P* > 0.05). Therefore, the MH + IGF-MNDM can reduce the excessive inflammatory response induced by TBI during the acute phase and help improve neurological prognosis following TBI.

## Discussion

Dural defects caused by TBI are a major clinical issue. The complex pathological microenvironment following TBI presents challenges for nerve repair and regeneration, which impacts the patient’s recovery of cognitive and motor abilities (Risling et al., 2019; Buhlman et al., 2021; Wang et al., 2024). Appropriate intervention can promote the survival or proliferation of damaged neural cells at different stages after TBI, thereby protecting nerve function and laying a solid foundation for subsequent recovery following TBI (Ma et al., 2021; Fu et al., 2022; Chen et al., 2023).

During the acute injury stage after TBI, the excessive expression of inflammatory factors hinders nerve survival and increases the severity of nerve damage (Kalra et al., 2022). Repair after brain injury is a prolonged process, and secondary nerve injury, such as oxidative stress and ischemia-reperfusion, will continuously destroy nerve cells and hinder their repair (Fesharaki-Zadeh, 2022). Therefore, the acute phase of TBI requires anti-inflammatory intervention to block the effects of the inflammatory cascade on neural survival. Moreover, establishing a long-term treatment strategy to relieve secondary injury following TBI and provide continuous repair for injured nerves is crucial. Previous studies have shown that the electrospun artificial dural drug delivery system has limitations in the time regulation function. Meeting the treatment needs at different time points following TBI is challenging, and it is impossible to achieve varied dual drug release during the acute and long-term stages (Shahriar et al., 2019; Buhlman et al., 2021). Given the varied drug intervention requirements at different TBI stages, we encapsulated MH within the shell layer of dual-layered core-shell nanofibers. The rapid release of MH during the early phase of TBI promotes the polarization of BV2 cells from the pro-inflammatory M1 phenotype to the anti-inflammatory M2 phenotype, which effectively regulates the excessive inflammatory microenvironment post-TBI. The results of the TBI rat model demonstrated that the dura mater significantly reduced the expression levels of pro-inflammatory cytokines. This mechanism may be related to the inhibition of the nuclear factor kappa-B signaling pathway activation by M2 microglia, which can downregulate the overexpression of pro-inflammatory factors, such as TNF-α and IL-1β (Jin et al., 2023; Zhang et al., 2023b). Indeed, M2 microglia play a neuroprotective role by secreting anti-inflammatory cytokines (Colonna and Butovsky, 2017; Song et al., 2019; Xiao et al., 2020). To achieve long-term nerve repair following TBI, a dual-layer encapsulation strategy was used to load IGF-1 into the core structure of the dual-layered core-shell nanofibers to enable long-term sustained release of IGF-1. Our results showed that the long-term release of IGF-1 from the dura mater played a key role in nerve repair and protection against primary and secondary nerve injury after TBI. This mechanism may involve the cascade activation of the IGF-1/phosphatidylinositol 3-kinase (PI3K)/protein kinase B (Akt)/B-cell lymphoma 2 (Bcl-2) signaling pathway. Activated Akt affects the expression of subsequent apoptotic signaling pathways by regulating the balance of Bcl-2-associated agonist of cell death/Bcl-2 apoptosis-related proteins and promoting the survival and repair of damaged neural cells (Zhang et al., 2017; Li et al., 2020). Our dual-layered core-shell nanofibers exhibited differential dual-drug release patterns for MH and IGF-1: the immune microenvironment was regulated during the early stage, and nerve repair and protection were facilitated long-term. This phase-adaptive intervention strategy effectively blocks the cascade of pathological processes associated with TBI and thus serves as an innovative solution for improving long-term neurological outcomes.

To effectively repair damaged nerves and reduce complications after brain injury, the dura mater needs to exhibit a variety of functions, such as antibacterial, anti-adhesive, anti-leakage, and barrier properties, that provide comprehensive protection and support (Lv et al., 2016; Gong et al., 2024; Aili et al., 2025). Previous studies have shown that currently available electrospun dura mater lack the integration of multiple functions, such as anti-adhesion, CSF barrier, and nerve regeneration, although numerous studies have reported their successful application in clinical practice (Park et al., 2018; Liao et al., 2021; Shafiq et al., 2025). This highlights the urgency of developing versatile biomimetic dural substitutes. Infection control following craniocerebral surgery is a key factor that affects prognosis (Sanpakitwattana et al., 2022). Our NDM showed good antibacterial capacity, which will help reduce surgical site infection and promote a favorable environment for nerve repair. For the prevention of postoperative adhesions, we introduced HA to reduce adhesion and excessive proliferation of fibroblasts. The PCL/HA-NDM exhibited a positive anti-adhesion effect and prevented tissue adhesion. Furthermore, the dense fibrous structure of the dura mater itself provided a physical barrier function for the cells. The dura mater effectively prevented the invasion of external fibroblasts, thereby avoiding the formation of tissue adhesions and fibrosis within the brain. This ensured a favorable environment for the repair of internal nerve cells.

CSF leakage may also cause serious infection and even death (Wang et al., 2023). The MNDM we constructed showed excellent anti-leakage performance under both dynamic and static solution conditions, which is conducive to maintaining the stability of internal pressure in brain tissue. In addition, given that the dura mater is a neurosurgical implant, a high standard of biocompatibility is crucial. *In vitro* and *in vivo* biocompatibility experiments revealed good biocompatibility of the dura mater, which demonstrated its safety and functionality as a physical barrier. Furthermore, the MH + IGF-MNDM possessed mechanical and degradation properties that allowed effective resistance to external pressure and offered the brain tissue considerable support and protection.

Although the prepared MNDM was effective in dealing with the complex pathological processes following TBI, the study has several limitations. First, the RB was used as a model drug to examine the drug release behavior of the dural scaffolds. However, RB does not directly reflect the IGF-1 release behavior of the dura mater. Therefore, we plan to further investigate the release behavior of IGF-1 in the future. Additionally, we preliminarily confirmed that the MNDM improves the inflammatory microenvironment and exerts a neuroprotective effect by promoting neurite regeneration and inhibiting apoptosis. However, we did not systematically investigate the specific mechanisms by which signaling pathways, such as nuclear factor kappa-B and IGF-1/PI3K/AKT signaling pathways, are regulated by the MNDM. Further experimental studies are needed to clarify the specific molecular mechanisms underlying the regulation of the inflammatory microenvironment and the neuroprotective effect of the MNDM following TBI.

In conclusion, we successfully developed an MNDM using innovative design and manufacturing methods and examined its reparative effects following TBI. The preparation of the dura mater with a differential dual-release mechanism using emulsion electrospinning and coaxial electrospinning technologies effectively promoted the repair of damaged nerves after TBI. The early release of MH by the dura mater regulated the inflammatory environment during the acute stage and increased the survival rate of damaged neural cells. Furthermore, the continuous release of IGF-1 enabled long-term repair and protection of damaged nerves. Additionally, the dura mater possesses multiple functional characteristics, such as antibacterial, anti-leakage, anti-adhesive, barrier properties, and good biocompatibility. Therefore, our MNDM has great potential for application to TBI and is expected to be used more widely in the future.

## Additional files:

***Additional Figure 1:*** *Photographs of each kind of nanofibrous dura maters and cross-sectional images of multilayered nanofiber dura mater.*

Additional Figure 1 Photographs of each kind of NDM and cross-sectional images of the MNDM
(A) Photograph of the PCL/HA-NDM. (B) Photograph of the PCL-NDM. (C) Photograph of the MH + IGF-NDM. (D) Cross-section of the MH + IGF-MNDM. The surface structure of each NDM was smooth, and no obvious droplets were observed. Scale bars: 10 mm. HA: Hyaluronic acid; IGF: insulin-like growth factor; MH: minocycline hydrochloride; NDM: nanofiber dural mater without drug; PCL: polycaprolactone.

***Additional Figure 2:*** *Fiber diameter distribution of each kind of nanofibrous dural fibers.*

Additional Figure 2 Fiber diameter distribution of each kind of NDM.
(A) PCL/HA-NDM. (B) PCL-NDM. (C) MH + IGF-NDM. HA: Hyaluronic acid; IGF: insulin-like growth factor; MH: minocycline hydrochloride; NDM: nanofiber dural mater without drug; PCL: polycaprolactone.

***Additional Figure 3:*** *Degradation behavior of MH + IGF-MNDM.*

Additional Figure 3 Degradation behavior of the MH + IGF-MNDM
(A) pH curve. (B) Mass decrease curve. All quantitative data are expressed as means ± standard deviations (*n* = 3). (C) Scanning electron microscopy images of the inner and outer dura mater 30, 60, and 90 days after degradation. After degradation on days 30, 60, and 90, the dura mater in each group maintained a stable microstructure. Scale bars: 10 μm (C). HA: Hyaluronic acid; IGF: insulin-like growth factor; MH: minocycline hydrochloride; NDM: nanofiber dural mater without drug; PCL: polycaprolactone.

***Additional Figure 4:*** *Effects of different concentrations of hydrogen peroxide on the morphology of SH-SY5Y cells.*

Additional Figure 4 Effects of different concentrations of H_2_O_2_ on the morphology of SH-SY5Y cells.
(A-G) 0, 25, 50, 100, 200, 400, 800 μM H_2_O_2_. When the concentration of hydrogen peroxide exceeded 200 μM, the number of cells in the field decreased, and the neurite length of SH-SY5Y cells shortened. Scale bar: 100 μm.

***Additional Figure 5:*** *Antibacterial effects of different kinds of dura maters against S. aureus.*

Additional Figure 5 Antibacterial effects of different kinds of dura mater against *S. aureus*
(A-E) Photographs of agar plates of *S. aureus* of the control (A), NDM (B), IGF-NDM (C), MH-MNDM (D), and MH + IGF-MNDM groups (E). (F) Antibacterial rates of different NDM against *S. aureus*. The MH + IGF-NDM and MH-NDM groups performed better than the other groups in resisting *S. aureus*. All quantitative data are expressed as means ± standard deviations (*n* = 4). ^***^P < 0.001 (one-way analysis of variance, followed by Tukey’s multiple comparison). IGF: insulin-like growth factor; MH: minocycline hydrochloride; NDM: nanofiber dural mater without drug; *S. aureus*: *Staphylococcus aureus*.

***Additional Figure 6:*** *Antibacterial effects of different kinds of dura maters against E. coli.*

Additional Figure 6 Antibacterial effects of different kinds of dura mater against *E. coli*.
(A-E) Photographs of agar plates of *E. coli* of the control (A), NDM (B), IGF-NDM (C), MH-MNDM (D), and MH + IGF-MNDM groups (E). (F) Antibacterial rates of different NDM against *E. coli*. The MH + IGF-NDM and MH-NDM groups performed better than the other groups in resisting *E. coli*. All quantitative data are expressed as means ± standard deviations (*n* = 4). ^***^P < 0.001 (one-way analysis of variance, followed by Tukey’s multiple comparison). *E. coli*: *Escherichia coli*; IGF: insulin-like growth factor; MH: minocycline hydrochloride; NDM: nanofiber dural mater without drug.

***Additional Figure 7:*** *NIH3T3 cell penetration on different kinds of dura maters after 3 (A) and 5 (B) days.*

Additional Figure 7 NIH3T3 cell penetration in different kinds of dura mater after 3 (A) and 5 (B) days.
All quantitative data are expressed as means ± standard deviations (*n* = 3). ^***^P < 0.001 (one-way analysis of variance, followed by Tukey’s multiple comparison). IGF: insulin-like growth factor; MH: minocycline hydrochloride; MNDM: multilayered nanofiber dural mater; NDM: nanofiber dural mater without drug; PCL: polycaprolactone.

***Additional Figure 8:*** *Fluorescent dye penetration experiment in MH + IGF-MDNM.*

Additional Figure 8 Fluorescent dye penetration experiment in the MH + IGF-MDNM.
Plots of cross-sectional fluorescence images 5, 10, 30, and 60 minutes after the dropwise addition of RB solution. The red fluorescence could not penetrate the lower surface of the MH + IGF-MDNM at any time point after the RB solution was added. Scale bar: 100 μm. IGF: Insulin-like growth factor; MH: minocycline hydrochloride; MNDM: multilayered nanofiber dural mater.

***Additional Figure 9:*** *Static leakage prevention ability of different kinds of dura maters.*

Additional Figure 9 Static leakage prevention ability of different kinds of dura mater.
(A) Images at the beginning at 0 hours. Image after 0 (B) and 20 hours (C) of inversion. No red droplets were found in any groups at 0 hours. Only the MH + IGF-NDM group had red droplets flowing down in the 1.5-mL inverted centrifugal tube at 20 hours. IGF: Insulin-like growth factor; MH: minocycline hydrochloride; NDM: nanofiber dural mater without drug.

***Additional Figure 10:*** *Dynamic leakage prevention ability of different kinds of dura maters.*

Additional Figure 10 Dynamic leakage prevention ability of different kinds of dura mater.
Water sealing properties of different NDM in 15-mL inverted centrifugal tubes. There was no leakage of aqueous solution after severe dural vibrations in any group. HA: Hyaluronic acid; IGF: insulin-like growth factor; MH: minocycline hydrochloride; MNDM: multilayered nanofiber dural mater; NDM: nanofiber dural mater without drug; PCL: polycaprolactone.

***Additional file 1:*** *Open peer review report 1.*

OPEN PEER REVIEW REPORT 1

## Data Availability

*All relevant data are within the paper and its Additional files.*
