# Supplementary material for: Complex bioactive nanofibrous dura mater repairs traumatic brain injury
Source: Neural Regen Res. 2025 Sep 29;21(9):4275–89. doi: 10.4103/NRR.NRR-D-25-00621 (PMC13561431; doi:10.4103/NRR.NRR-D-25-00621)
Supplement: Supplementary file 1 [file NRR-21-4275_Suppl1.pdf]

OPEN PEER REVIEW REPORT 1

Name of journal: Neural Regeneration Research
Manuscript NO: NRR-D-25-00621
Title: Complex Bioactive Nanofibrous Dural Substitutes Promote the Repair of Traumatic Brain Injury through Neuroprotection, Immunomodulation, Anti-leakage, and Anti-adhesion
Reviewer's Name: Esra Cansever Mutlu
Reviewer's country: UK

COMMENTS TO AUTHORS

Very nice paper, thank you. Just I saw a few mistakes.

1- Research has shown
that Minocycline (MH) garners significant attention for its prominent anti-inflammatory effects, coupled with antimicrobial properties[29-31]. (We need to see which authors and Year) ie Abc., E. et al. 2025 has shown.

2- Regarding to release profiles, 1/2h gives you free-drug which was not incorporated to your material. You have not done that. Anyhow, We need to see release profiles with log curve hourly. Additionally, UV curve that includes equation.

3- Antibacterial activity is a big claim for this paper with just S. Aerus. We need to see at least E Coli, P Aeruginosa and (maybe Candida Albicans?) must be done! (at least) for antimicrobial property. If not, please indicate its importance in somewhere in conclusion part, just telling which should had been added to mention despite the fact just one bacterial response.
